# NBS/NIST Gas Thermometry From 0 to 660 °C

**DOI:** 10.6028/jres.095.028

**Published:** 1990

**Authors:** J. F. Schooley

**Affiliations:** National Institute of Standards and Technology, Gaithersburg, MD 20899

**Keywords:** gas thermometer, IPTS-68, thermodynamic temperature, thermometry

## Abstract

In the NBS/NIST Gas Thermometry program, constant-volume gas thermometers, a unique mercury manometer, and a highly accurate thermal expansion apparatus have been employed to evaluate temperatures on the Kelvin Thermodynamic Temperature Scale (KTTS) that correspond to particular temperatures on the 1968 International Practical Temperature Scale (IPTS-68). In this paper, we present a summary of the NBS/NIST Gas Thermometry project, which originated with planning activities in the late 1920s and was completed by measurements of the differences *t*(KTTS)-*t*(IPTS-68) in the range 0 to 660 °C. Early results of this project were the first to demonstrate the surprisingly large inaccuracy of the IPTS-68 with respect to the KTTS above 0 °C. Advances in several different measurement techniques, development of new, specialized instruments, and two distinct sets of gas thermometry observations have resulted from the project.

## 1. Introduction

The terms “thermodynamic temperature” and “absolute temperature” are used synonymously to refer to the parameter that occurs in many theoretical equations in physics and chemistry. By the middle of the 19th century, Carnot, Kelvin, and others had elucidated the principles of thermodynamics, and Kelvin had devised a universal scale of temperature that could be shown to be independent of the properties of any particular material [1]. His scale was very general, depending only upon the efficiency of Carnot’s ideal heat engine; a natural consequence of his idea was the existence of an absolute zero of temperature defined by the absence of any heat energy in the substance under test.

Kelvin suggested that his scale could be particularized by specifying a number value for some reproducible state of matter; taking note of contemporary work on the thermal expansion of gases between 0 and 100 °C (the freezing and boiling points of water on the centigrade scale that was then in common use), he suggested that a workable thermodynamic scale could be obtained by adding 273 to all temperature values obtained by use of that centigrade scale.

The fact that the thermal expansion of gases was an important topic in 19th century science made the gas thermometer a natural choice for realizing Kelvin’s new thermodynamic temperature scale. Measurements of thermodynamic temperature over a wide range can be performed with reasonable accuracy by use of gas thermometers, providing that proper account is taken of the properties of the real substances used in the experimental work. In principle, the ideal gas law,

PV=nRT, (1)

where *P* represents the thermodynamic pressure, *V* stands for the volume of a bulb that contains *n* moles of an ideal gas, *R* denotes the gas constant, and *T* indicates the thermodynamic temperature, can be employed to realize the KTTS through the use of gas thermometers of various types. For instance, the unknown KTTS temperature *T_u_* of a constant-pressure or a constant-volume gas thermometer can be evaluated approximately in terms of the Celsius reference temperature *t*_r_ by using the equation

$$T_{u}=\left(t_{u}+273\right)=\left(t_{r}+273\right)\left(V\left[P,t_{u}\right]/V\left[P,T_{r}\right]\right)$$ (2)

or

$$T_{u}=\left(t_{u}+273\right)=\left(t_{r}+273\right)\left(P\left[V,t_{u}\right]/P\left[V,t_{r}\right]\right),$$ (3)

respectively. Kelvin’s original constant additive term, 273, approximately converts Celsius scale temperatures to thermodynamic Kelvin temperatures. In the constant-pressure case [eq (2)], the Kelvin reference temperature is multiplied by the ratio of volumes that contain a fixed number of moles of an ideal gas at a fixed pressure and at the unknown and reference temperatures, respectively, to produce a value of the unknown Kelvin temperature. In the constant-volume case [eq (3)], the multiplicative factor is the ratio of pressures at constant volume and at the two respective temperatures.

It is not difficult to discover that the simple equations given above apply only at a very elementary level of accuracy. For example, the present definition of the Kelvin Thermodynamic Temperature Scale assigns the value 273.16 K to the temperature of the triple point of water, so that the constant in eqs (2) and (3) must be modified accordingly. In addition, substantial corrections must be made to account for the facts that real gases do not behave ideally over any considerable range of temperature, so that so-called “virial corrections” must be incorporated into the equations; that gas bulb materials undergo thermal expansion as well as dilation or contraction from any pressure differences that exist across the bulb walls, changing the actual volumes of the gas bulb; that “dead space” volumes inevitably enter gas thermometric apparatus and must be properly considered; that accurate pressure and volume measurements can be made only with great difficulty; and that laboratory instrumentation generally can be calibrated only within certain limits. As a result of these problems, the evaluation of thermodynamic temperatures by gas thermometry has always been uncertain, with 0.01% representing the limiting accuracy until the work of Guildner and Edsinger established a new level of accuracy for this method. Berry [2] and Kemp et al. [3] have since accomplished gas thermometry of similarly high quality to elucidate the KTTS in the range below 273 K. Beattie [4] has given a thorough discussion of the experimental problems inherent in performing accurate gas thermometry.

## 2. Principles of the NBS/NIST Gas Thermometer

Very little has been written about the origins of the NBS gas thermometry program. Although Guildner et al. [5] refer to early developments in precision manometry for gas thermometers at NBS by Cragoe, Godfrey, Meyers, and Thompson, only internal NBS reports [6] contain information about NBS gas thermometry studies prior to the brief description given by Stimson during the Third Temperature Symposium in 1954 [7]. These internal reports date from 1928; they reflect, even then, efforts to employ many of the primary features of the present NBS gas thermometer—the use of a mercury manometer for accurate measurements of the pressure ratios arising in the use of a constant-volume gas thermometer as given in eq (3), the use of gage block end standards to support the reference cell of the manometer, and the careful isolation of the gas thermometer system from both mechanical and thermal disturbances.

**Figure 1:**
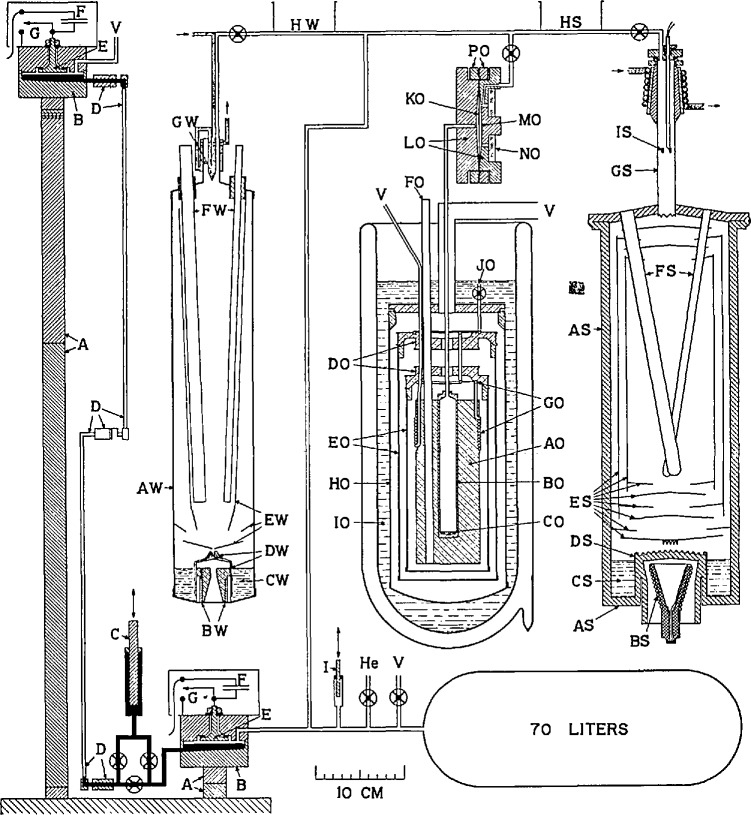
Diagram of NBS thermometer calibration apparatus used during the early 1950s. The diagram shows, from the left, a precision manometer that is similar in many respects to the one used in all of the NBS/NIST gas thermometry measurements, a steam-point boiler, an oxygen-point apparatus, and a sulfur-point boiler. A—End standard gage blocks, intended to be wrung together to define the cell separation. B—Large-meniscus (~7.3 cm inside diameter) mercury cells. C—Mercury pump for the adjustment of the mercury levels in the manometer. D—Movable mercury lines with rotating joints. E—Fixed capacitor plates for mercury level sensing. KO, LO, MO—Components of a capacitance diaphragm gage used to separate the helium gas used in the manometer from the oxygen used in the oxygen-point apparatus. (Reprinted from reference [7].)

Most of the significant features of the manometer used in the present-day NBS gas thermometer can be observed in figure 1, taken from reference [7]. Although this figure actually portrays an NBS thermometer calibration facility dating from the early 1950s, Stimson notes that the manometer shown at the left of the figure was designed for precision gas-thermometer measurements. The manometer was contained in a mechanically isolated, temperature-controlled room. The measuring and reference mercury surfaces were placed in large, carefully designed cells. They were connected by a small mercury line that contained swivel joints to permit the raising and lowering of the upper cell. The mercury levels in both cells were sensed by capacitor plates. (Initially, interferometric sensing of the mercury levels was considered for the level-sensing, but the existence of ripples on the mercury surfaces—which never have been completely eliminated—spoiled the sensitivity of that method. In contrast, the use of capacitance level-sensing was shown to “average out” standing waves on the uneven mercury surface rather accurately.) Since the mercury surfaces in the upper and lower cells could be maintained at very nearly fixed, equal distances from the bases of the cells, the height of the mercury column in any measurement would depend only upon the height of the stack of end standard gage blocks and the verticality of the stack. Accurate knowledge of the overall pressure generated in the manometer depended as well, of course, upon accurate knowledge of the density of the mercury in the manometer and of the value of the gravitational constant at the location of the instrument. In turn, the “fixed, known density” requirement placed tight restrictions upon the cleanliness of the manometer system and upon regulation and measurement of its temperature.

Besides the specialized manometer system, figure 1 shows, in conjunction with the oxygen boiling-point apparatus, the use of a metal diaphragm to separate the manometer’s working gas from the gas employed in the test environment. Once again, electrical capacitance was employed to sense position; the deviation of the differential pressure across the diaphragm was measured in terms of changes from the null-point capacitance between the diaphragm and a fixed reference plate in its casing.

In 1955, Stimson [7] estimated the levels of uncertainty that reasonably could be expected in the use of the NBS manometer. These include uncertainty in the density of mercury, about 20 ppm; in the gravitational constant, about 10 ppm, and in the end standards, about 2 ppm.

The original plan for the NBS gas thermometer was to measure the ratio of the pressures generated in the gas thermometer at two temperatures, one of which would approximate the ice point (later refined to the more precise triple point of water). By fixing the number of moles of gas involved and using the ratio technique, Stimson and his colleagues planned to employ a more advanced form of eq (3) to evaluate the unknown temperature in thermodynamic terms, relative to the first. Initial NBS discussions probed the possibility of using a gas-bulb material made of an alloy whose thermal expansion might be nearly zero over a limited range of temperature. They also considered the relative advantages of utilizing a working gas of known non-ideality, versus the technique of making measurements over such an extended range of filling pressures that an extrapolation to zero pressure might be valid.

## 3. Present Manometer and Its Uncertainties

The present NBS/NIST gas-thermometer manometer became a reality following the addition of L. A. Guildner, R. E. Edsinger, and R. L. Anderson to the NBS staff during the late 1950s [8]. Many refinements of the original manometer were incorporated into later versions by this group, as well as one completely new feature—a second lower mercury cell that served to evaluate the verticality of the gage-block stack.

The first detailed discussion of the components of the manometer system was published in 1965. This paper [9] noted the value of transformer ratioarm bridges with three-lead capacitors for the precise measurement of capacitance, and it provided details of the construction and wiring of such a bridge as it applied to sensing the mercury level in a manometer and to determining the inside diameter of a relatively long metal capillary.

**Figure 2:**
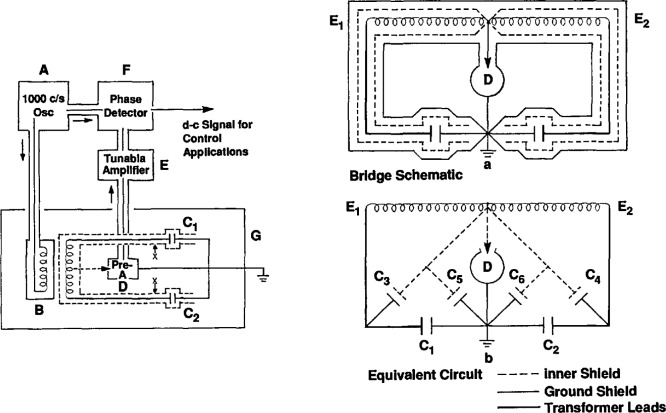
Left side—Block diagram of the capacitance bridge used to match the mercury levels in the gas thermometer manometer. Right side—Capacitance bridge schematic, shown without the primary windings (upper drawing), and its equivalent circuit (lower drawing). E_1_, E_2_—voltages opposite in phase, and with a ratio that is fixed within I ppm of the turns ratio. C_1_, C_2_—active capacitor elements. C_3_, C_4_—minimized capacitance between the inner shield and the transformer leads. C_5_, C_6_—minimized capacitances between the inner shield and the ground shield. D—High-impedance detector. (Reprinted from reference [9].)

A schematic drawing of the capacitance bridge appears in figure 2. The bridge was especially designed to permit use of two test capacitors with one plate of each grounded, as would be the case in the manometer application. A double-shielding arrangement facilitated this design. In addition, elimination of the effects of capacitive coupling between the primary and secondary windings was achieved by the introduction of two electrostatic shields. Attention was also given to the problems involved in comparison measurements with a reference capacitor. The estimated sensitivity of the bridge was 1×10^−5^ pF. The manometer pressure uncertainty arising from its use was estimated as not larger than 0.15 Pa.

**Figure 3:**
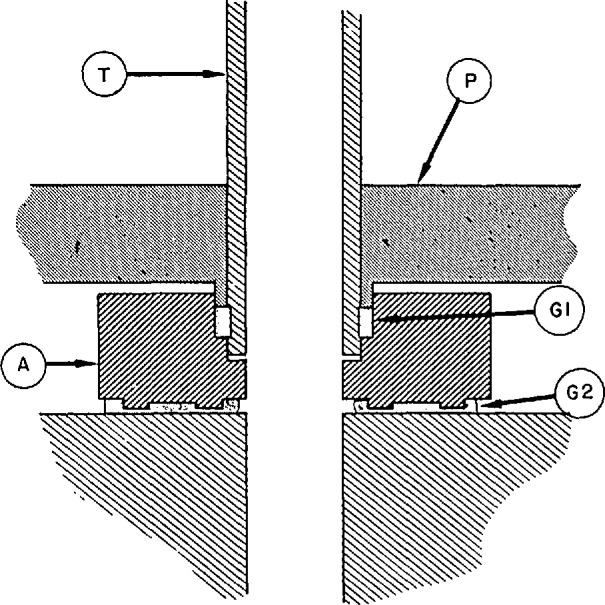
Details of two types of captured-elastomer seals used in the NBS/NIST manometer system. Ring A is fabricated with a land and groove on its bottom face and a compound receptacle for a locking seal on its top surface. Tube T is grooved to receive a polytrifluorochloroethylene gasket G1 which is then compressed by the force of a pusher P. The pusher also compresses polytetrafluoroethylene gasket G2. The total effect is to lock the tubing into a demountable assembly that both seals and locks it in place. (Reprinted from reference [10].)

A technique for utilizing fluorocarbon elastomers for high-vacuum seals in several articulating-arm and demountable-seal systems throughout the gas thermometer was discussed in a separate paper [10]. The seals were based upon the principle of stressing the fluorocarbon beyond its yield point; then it behaves as a highly viscous fluid, accommodating to mating surfaces even of relatively poor quality, and it also becomes much less porous, preventing leaks larger than 2×10^−10^ cc/s. Figure 3 shows the critical features of this type of seal. Poly-tetrafluoroethylene, which has a rather small coefficient of friction, was used in seals in which the component parts were intended to move, whereas polytrifluorochloroethylene was used in locking seals.

A full description of the manometer was published [5] in 1970. By that time, the instrument in its final form was installed and operating in the Gaithersburg gas thermometry laboratory of the NBS.

**Figure 4:**
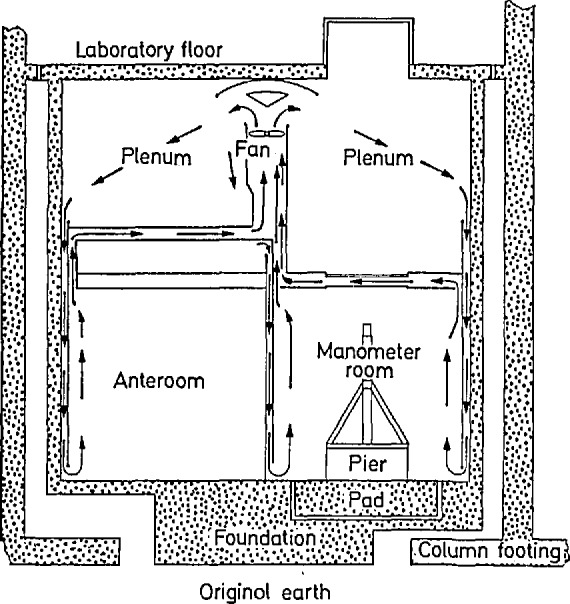
Schematic cross-section of manometer cellar beneath the NBS/NIST gas thermometer laboratory. The manometer is mounted upon a concrete pier that was made separate from the laboratory building to minimize vibration. A dedicated air conditioning system, vigorous air circulation, multi-point air-temperature sensing, and carefully designed controllers maintain millikelvin stability in the cellar temperature. The anteroom is used to temper and store a collection of end standard gage blocks and as a toolroom. (Reprinted from reference [5].)

The manometer was installed in a specially prepared room below the basement of the Physics building at the new site. Figure 4 shows in schematic form a cross section of this two-level cellar, which provides protection from electromagnetic interference, from temperature inhomogeneity beyond the millikelvin level, and from most sources of vibration. The air conditioning flow moves through a multi-ducting system that incorporates temperature-controlled dampers; the relative humidity is held at 50% (referred to a temperature of 20 °C), and the temperature can be held constant within 2 mK/d.

The manometer itself was installed on a freestanding pier with a cap consisting of a sheet of steel 0.86 m square and 7.6 cm thick. An Invar^1^ bar, resting upon knife edges, supports the two lower mercury cells and the gage block stack that in turn supports the upper cell. Great care was taken to provide flat, co-planar (within 0.1 *μ*m) surfaces atop the Invar bar onto which could be wrung the cells and gage blocks. Provision was made for pumping a vacuum in axial holes in the gage blocks as a check on the quality of the wringing. The temperature of the manometer is measured by a dedicated platinum resistance thermometer, while the stability of the articulating-arm system is determined from measurements of a set of thermocouple thermometers arrayed along the arms. Operation of the manometer can be accomplished from the main laboratory control panel.

**Figure 5:**
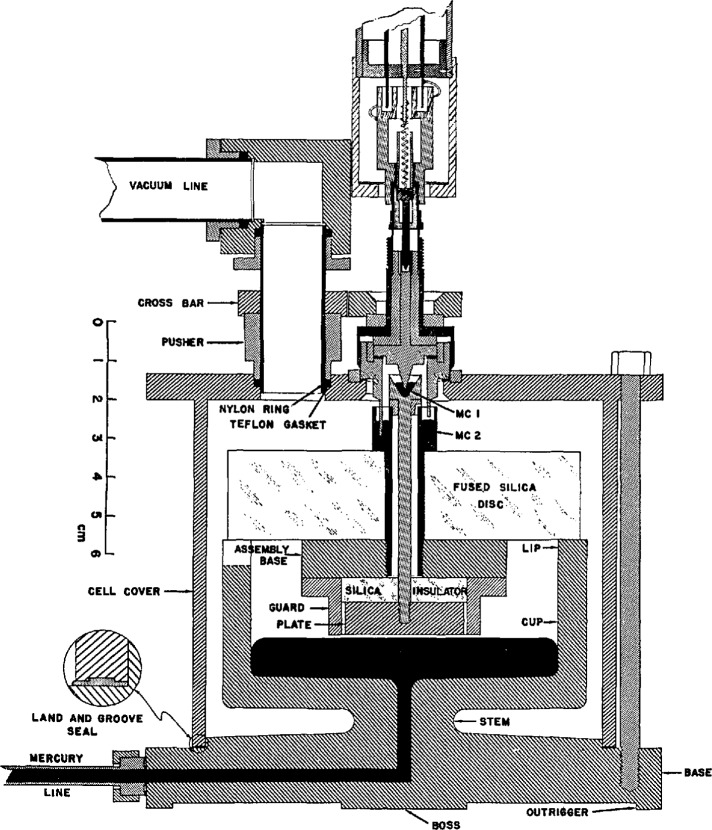
Cross-section of upper mercury manometer cell, showing details of the three-lead capacitance level measurement and the seals. The mercury cup was designed to maintain a stable position independent of pressure, and to be wrung to a gage block at the bottom and to a fused silica mounting disc at the top. Wringing the components of the capacitance level-detection assembly to the fused silica disc minimized the likelihood of drift in the mercury level, as well as deviations from co-planarity between the mercury surface and the capacitor plate. Mercury contacts MC1 and MC2 minimized stress arising from stiffness in the electrical contacts. (Reprinted from reference [5].)

Details of the capacitance level-sensing and other techniques used in the construction of the mercury cells are illustrated in figure 5. The flatness of the mercury meniscus, the effect of ripples on the measured height of the mercury, and the stability of the mercury level afforded by this technique are discussed in some detail.

Also discussed in the 1970 paper are the components of uncertainty that make up the overall accuracy of the manometer when it is used as a part of the gas thermometer in the range 10–130 kPa (0.1–1.3 atm). In table 1 is given a short summary of this analysis. Note that the overall uncertainty of the manometer as a pressure standard is approximately 2 ppm; the uncertainty drops to 1.5 ppm if the manometer is used to compare pressures.

## 4. First NBS Gas Thermometer

In its first version, the gas-bulb/thermostat assembly of the NBS/NIST gas thermometer had been intended to consist of a spherical gas bulb with a heavy protective casing, enclosed by a multi-layered, electrically heated furnace. The furnace was to be placed above the manometer connection [8], The spherical shape is a strong one, and it permits relatively simple thermal expansion corrections to the bulb volume. The protective casing would allow a thin layer of gas at the working pressure to surround the gas bulb as a guard against pressure deformation of the bulb. Connecting the capillary to the bottom of the gas bulb and placing the assembly above the level of the pressure sensor would then minimize the likelihood of convective heat flow within the capillary, allowing it to be made slightly larger in diameter than would be the case if the temperature gradient were reversed. In practice, however, both the shape and the positioning of the thermometer were modified; machining and assembly of a protected spherical gas bulb and the establishment of a homogeneous temperature environment within a multi-shell configuration proved difficult. Therefore, a cylindrical bulb protected by a cylindrical casing and the use of stirred-liquid thermostat baths, entered from the top, were adopted.

The first working version of the NBS/NIST gas thermometer [11] made use of the manometer as described in the previous section. Gage blocks for use in the manometer were specially calibrated (with an estimated uncertainty of ±0.3 ppm) by the NBS Dimensional Metrology Laboratory. The acceleration due to gravity at the location of the manometer was measured by the U.S. Geological Survey (with an estimated uncertainty of ±0.5 ppm). A 5-m tube, with an accompanying set of thermocouple thermometers to establish its temperature profile, was installed to carry the manometer gas (^4^He) from the manometer cellar to the upper laboratory level. Remote control of the operating valves for opening and closing the mercury connections between the manometer cells from the upper laboratory was arranged. Finally, the capacitance bridge for monitoring the mercury levels (and a mechanism for slightly adjusting those levels) was installed in the upper laboratory. Thus, once the gage blocks appropriate for the production of a desired pressure were chosen and assembled into the manometer, final checking and adjustment of both the mercury level and the working gas pressure could be performed from the upper laboratory without disturbing the temperature in the manometer cellar.

The manometer was separated from the gas thermometer bulb by the diaphragm of a capacitance diaphragm gage, by a set of constant-volume valves, and by a capillary tube. The gas bulb could be lowered by a hydraulic mechanism into any of several movable variable-temperature baths; both the bulb and its connecting capillary were protected from exposure to the bath liquids by protective casings made of Inconel.

**Figure 6:**
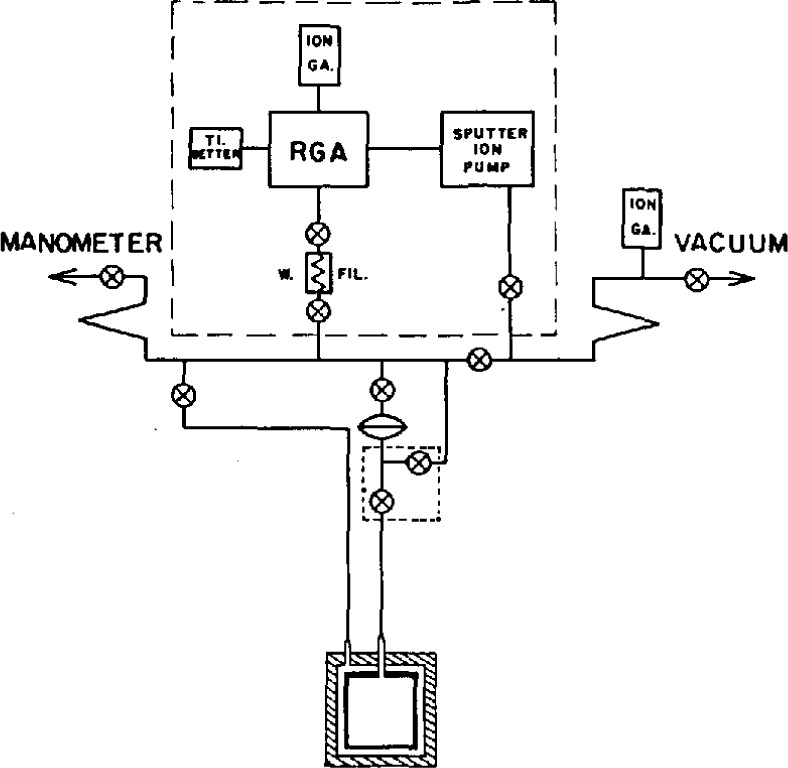
Elements of the first NBS/NIST gas thermometer. The gas bulb, a right circular cylinder of volume about 450 cm^3^, was made from sheets of a platinum-rhodium alloy. It was surrounded by a heavy casing of Inconel. Constant-volume valves and a capacitance diaphragm gage separated the gas bulb from the manometer and from a gas purification and analysis system composed of a tungsten filament, a titanium getter, and a residual gas analyzer pumped by an ion pump. (Reprinted from reference [11].)

A schematic diagram of the gas bulb, its protective casing, and a portion of the gas handling and analysis system is shown in figure 6. The gas bulb was a relatively thin-walled (0.94 mm sides and bottom, 2.79 mm top) right circular cylinder of Pt-Rh alloy; its volume was about 430 mL. Most versions were constructed from sheets of a single alloy (80 Wt% Pt, 20 Wt% Rh) or (88 Wt% Pt, 12 Wt% Rh), although in one case, the bulb inadvertently was fabricated from two different alloys. The working gas in all cases was ^4^He. Use of a residual gas analyzer allowed the evaluation of the purity of the working gas at a level sufficient to improve markedly the accuracy of the gas thermometer.

**Figure 7:**
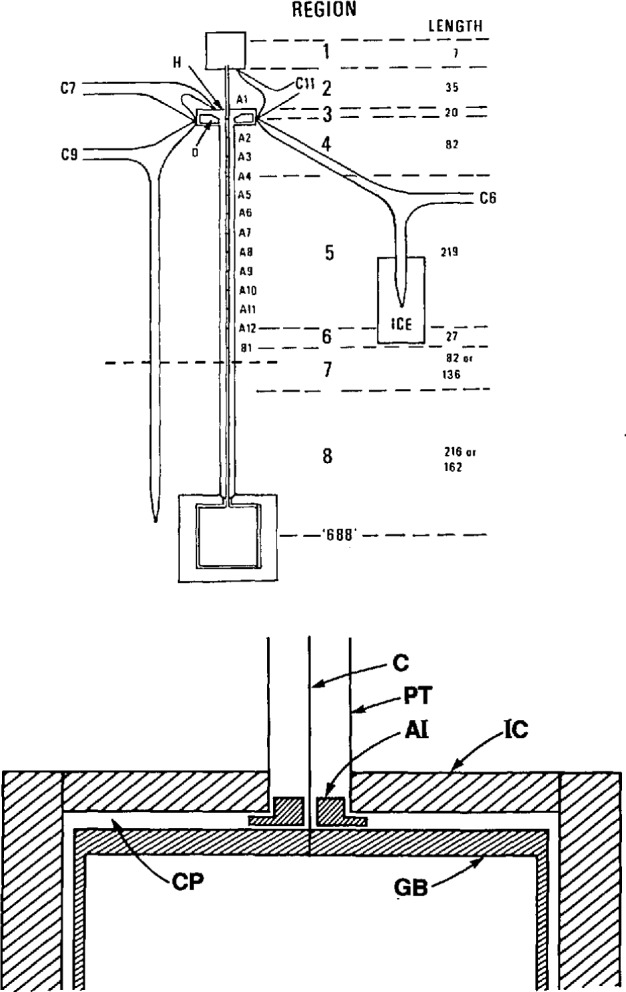
Schematic drawings of the NBS gas thermometer gas-bulb assembly and the capillary temperature-profile measurement system used with the stirred-liquid thermostat baths. The upper portion of the figure, reprinted from reference 21, shows the gas bulb and capillary with their protective casings. Sliding seal H allowed for the difference in thermal expansion between the capillary and its protective case. The drawing also shows the location of Pt wires welded to the capillary to form Pt vs. (Pt+10 Wt% Rh) thermocouple thermometers A1-A12 and B1, used to determine the temperature distribution along the capillary. The lower portion of the figure shows details of the gas thermometer construction in the region of the connection between the gas bulb GB and the capillary C, both made of Pt-Rh alloys. The capillary protection tube PT and the gas-bulb protective casing IC were made of Inconel alloy. AI denotes an alumina collar used to insulate the bulb electrically from the case and to create a gap CP between them. The gap, known as the counterpressure space, was filled with ^4^He gas held at the gas-bulb pressure. Both the bulb and case were constructed by welding, using the heliarc technique.

The designs of the gas bulb, the capillary, and the protective casing were intended to provide several results, as shown in figure 7. The Pt-Rh alloy composition was chosen for its uniform thermome-chanical properties, its high-temperature stability, and its stiffness relative to that of pure platinum. As mentioned above, the cylindrical shape of the bulb allowed for closer tolerances in construction and for greater ease in assembly than the spherical shape first considered. The flat top of the bulb was made of 2.8-mm stock to give it reasonable strength against deformation; by making the sides and bottom of thinner material (~ 1 mm) than the top, one could achieve a faster thermal response and a lighter weight to be suspended from the top. The capillary was made of approximately 1.6 mm o.d., 0.9 mm i.d. tubing of 90 Wt% Pt/10 Wt% Rh alloy. Use of that alloy for the capillary itself permitted the formation of a series of Type S thermocouple junctions along the capillary by simply welding Pt wire to the tube at appropriate intervals, and insulating the gas bulb, the capillary, and the wires from the Inconel protecting case and tube. Provision was also made for the difference in thermal expansion between the capillary and its Inconel protection tube. The gas bulb was isolated from the thermostating bath fluids and from pressure imbalances across its wall by a close-fitting, heavy-walled Inconel case. The wall of the protective case provided wells for four standard platinum resistance thermometers. The helium working gas was purified by passing it through molecular sieve material held at 78 K; its purity was evaluated through the use of a residual gas analyzer. As mentioned above, the working gas was separated from the manometer gas, also ^4^He, by a brass diaphragm whose position was monitored by a capacitance plate, forming a capacitance diaphragm gage. With its use, measurement of the pressure null was found to be accurate within approximately 4 mPa (0.03 *μ*m Hg).

Details of an application of the three-lead capacitance bridge (see sec. 3) employed to minimize the uncertainty of the dead-space correction in the gas thermometer were given in a 1971 paper [12]. In this application, the average diameter of the capillary tubing (see fig. 7) used to communicate the gas-bulb pressure to the manometer could be measured accurately within ±6×10^−5^ cm. The measurements were accomplished before assembly by the introduction into the capillary of a carefully designed probe; the capacitance between the probe and the tube could be measured as a function of the tube length. This information, coupled with the temperature profile obtained by the use of the thermocouples shown in figure 7, permitted detailed calculation (at ~l-mm intervals) of the capillary dead-space correction to be made. The method used to compute the dead-space correction from this source follows. The total quantity of gas in the gas thermometer during any particular measurement, *n*_0_, can be separated into two portions

$$n_{0}=n_{b}+n_{\mathrm{ds}},$$ (4)

where *n*_b_ denotes that portion of the gas that is contained in the gas bulb itself and thus is maintained at a uniform temperature, and *n*_ds_ denotes the portion of the gas that is contained in the “dead space,” that volume of the gas thermometer that experiences a temperature different from that of the gas bulb. The dead space in the NBS/NIST gas thermometer comprises the capillary up to the first valve, as shown in figure 6. Although it might be expected that the dead space would include one side of the capacitance diaphragm gage and the tube that connects it to the first valve, such need not be the case. If the pressure of the gas above the first valve is made equal to the pressure below the valve whenever it is to be opened, then no gas will pass through the valve during a set of measurements, and the gas thermometer may be considered to terminate at the first valve. In fact, this technique was used consistently throughout the NBS/NIST Gas Thermometry program.

For the capillary gas

$$n_{\mathrm{ds}}=\left[P_{0}/R\right][\sum_{k}\left\{V_{k}\left(1+\beta_{k}t_{k}\right)\theta_{k}\right\}].$$ (5)

In eq (5)*P*_0_ denotes the gas pressure at the capacitance diaphragm gage above the gas bulb, *R* stands for the gas constant, *V_k_* represents the volumes at 0 °C of approximately 1-mm segments (see fig. 7) of the capillary as measured capacitatively, *β_k_* indicates the average volume thermal expansion of the capillary material from 0 °C to *t_k_*, the Celsius temperatures of the capillary segments that are determined from measurements on thermocouples A1–B10, *θ_k_* indicate Celsius-to-Kelvin temperature conversion factors, and the summation occurs over the length of the capillary from the gas bulb up to the first valve.

**Figure 8:**
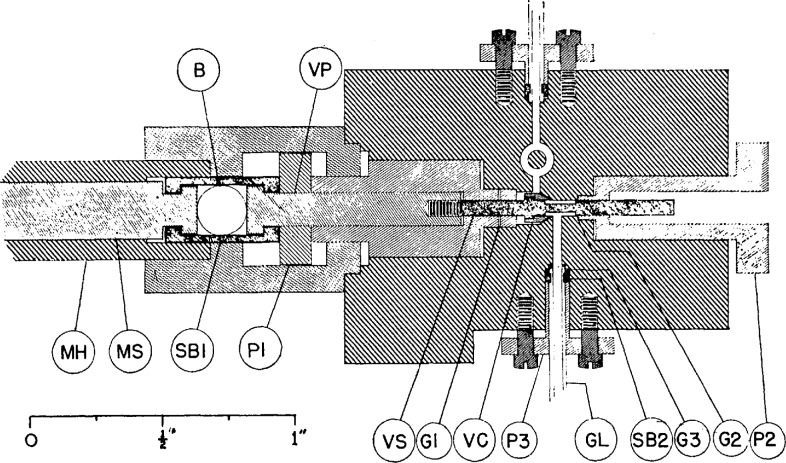
Cross section of constant-volume valve. A micrometer head, MH, is graduated in units of 0.0001”. It moves the spindle, MS, which in turn moves a steel ball, B, a push rod, VP, and the valve stem, VS. The valve cone, VC, was made with an included angle of 58 ° so that it would seal near its tip into a 60 ° seating hole. The stem is made uniform within ±0.00013 mm in diameter in order to ensure constant volume within 0.005 mm^3^. (Reprinted from reference [13].)

In order to restrict the effective dead space of the gas thermometer to the capillary, as described above, it was necessary to design a small, constant-volume valve to separate the gas bulb from the pressure-measurement system. The aim of the NBS workers was to limit the uncertainty in the ratio of the dead-space volume in the valve system to the volume of the gas bulb to 1 ppm. To accomplish this purpose, Anderson and Guildner designed a valve whose volume is constant within 0.1 mm^3^ at any position [13]. Details of the valve design are shown in figure 8.

Carefully designed, stirred liquid baths capable of temperature homogeneity at the level of a mil-lidegree or so at temperatures up to 500 °C have been described by Beattie [14]. Baths of similar design were built for the NBS/NIST gas thermometer. Each bath was enclosed in a large, heavy container that was fitted with wheels. The bath enclosure rested upon firebrick and was encircled by a helically coiled outer heater wire. Mineral insulation retarded radial heat flow to the outer shell. A steady voltage was applied to the outer heater at such a level that the bath temperature reached 2–3 °C below the desired value. A regulating heater took the form of a second helical coil that surrounded a central flow-directing cylinder that was open at both ends. At the bottom of the cylinder, a high-speed stirrer circulated the bath liquid, which flowed upwards past the gas thermometer assembly, outwards to the edge of the bath, and downwards to the bottom again. The gas thermometer assembly and the platinum resistance thermometers were thus continually bathed by the tempering bath liquid. The baths built with this design provided stable temperature environments for the gas bulb; the measured gas thermometer temperature was found to remain steady over a typical 1-week period within 0.4 mK, and within 0.04 mK over a typical 2-h period. Use of this type of thermal environment for the gas thermometer bulb, however, necessitated the construction of a hydraulic lifting system for the bulb assembly and the installation of articulating vacuum joints for the working gas lines. The latter were made similar to those employed in the manometer.

## 5. Scale Differences From 0 to 142 °C

After flushing the gas bulb with purified helium, it was necessary to spend a full month pumping the bulb to remove contaminating gases before commencing a set of gas thermometer measurements. After such a vacuum bakeout, however, a week’s isolation of the gas bulb resulted in a pressure rise of only 0.03 Pa. In preparation for a run, the gas bulb was filled with purified helium gas at the highest temperature to be measured, its pressure was verified over a three-hour period, and the bulb temperature on the IPTS-68 was recorded by three platinum resistance thermometers (PRTs) prior to reducing the bulb temperature to a lower unknown temperature or to the reference value near 0 °C. In these first experiments, six different filling pressures were used for each KTTS temperature that was to be determined. By plotting the apparent gas thermometer temperature as a function of pressure, Guildner, Anderson, and Edsinger could extrapolate the results to zero pressure with confidence, obviating the need for highly accurate second-virial-coefficient data on the ^4^He working gas.

The care with which the gas bulb and the gas purification and handling systems were prepared, together with the availability of sensitive in-line gas analysis equipment, paid immediate dividends as the first determinations of the steam-point temperature relative to the ice point were completed [11].

Three different gas bulbs were employed in this first series of measurements; the third was constructed so as to permit vacuum bakeout at temperatures as high as 800 °C. The KTTS temperature corresponding to the steam point as determined from the gas thermometer was found to decrease noticeably as the gas bulb cleanup proceeded. Eventually, the results stabilized. At that juncture, the steam point registered 99.973 °C on the thermodynamic Celsius scale, indicating that the International Practical Temperature Scale of 1968 was too high by 0.027 °C.

Because the Gas Thermometry group had not measured the thermal expansion of the metal from which the gas bulb had been constructed, and because they were not yet convinced that they had effectively eliminated the problem of sorption of impurity gases, they refrained from attaching an overall uncertainty to this first determination of the steam point. Nevertheless, it was apparent that the IPTS-68 might not have been as close an approximation to the Kelvin Thermodynamic Temperature Scale as had been generally thought (the estimated thermodynamic uncertainty of the IPTS-68 at the steam point had been assigned—conservatively, it had been supposed—as ±0.005 °C when the scale was formulated). Also apparent was the importance of cleanliness and the elimination from thermodynamic gas thermometry of sorbable impurities, which show such deviations from ideality that their presence had noticeably compromised most of the earlier work of this type.

After the gas thermometer measurements at the steam-point temperature were completed, Guildner and Edsinger [15] quickly introduced a new thermostated bath in which the fluid was a solution of water and potassium Chromate. With this bath, they extended the study of the differences between the KTTS and the IPTS-68 to cover the range 0 to 142 °C.

In completing this portion of the study, they also improved the method of purifying the ^4^He working gas: the gas was made to pass through a trap containing Ti chips held at 900 °C, where chemically reactive substances would combine with the Ti to form non-volatile compounds and, in some cases, hydrogen gas; then through a CuO trap, also held at 900 °C, to convert the hydrogen to water; and finally through a cooled (78 K) zeolite trap, to adsorb the water. They also arranged to allow the ion pumps to be valved off from the gas system to avoid possible contamination by outgassing when the ion pumps were turned off. As before, pressureratio measurements were made at several gas filling pressures, allowing extrapolation of the results to zero pressure.

The differences between the derived values of the Kelvin thermodynamic scale and the IPTS-68 over the range 0 to 142 °C were expressed [15] as

$$\begin{array}{l} t\left(\text{KTTS}\right)-t\left(\text{IPTS-}68\right)=-1.57478\times 10^{-4}t\\ -2.04508\times 10^{-6}t^{2}+6.08088\times 10^{-9}t^{3}{}^{\circ}C. \end{array}$$ (6)

The thermodynamic value of the steam point was given as 99.970±0.0035 °C at the 99% confidence level. The authors noted that, since it had been desired to maintain a truly centigrade International Temperature Scale (i.e., to maintain the ice and steam points exactly 100 Celsius degrees apart), then the ice point temperature should have been given the value 273.23 K rather than 273.15 K when the IPTS-68 was prepared. They also acknowledged that their present and future results on the KTTS could be incorporated into a table or equation of differences between that scale and the International one, rather than by replacing the International Scale with one that more closely approximates thermodynamic temperatures.

## 6. Second Version of the NBS/NIST Gas Thermometer

As a result of the experience gained in measurements to 142 °C, many other changes were made in the NBS/NIST gas thermometer and in the procedures for its use. Some of the changes were concerned with the problems of making measurements at still higher temperatures; others resulted from a desire to improve the quality of the observations.

Among the changes in equipment were the following:

- improvements in the ^4^He pumping system, both in capacity and flexibility;
- new thermostated bath for gas-bulb temperature equilibration;
- addition of an ac resistance bridge for PRT measurements and a digital voltmeter for thermocouple emf measurements; and
- addition of a thermomolecular-pressure-effect measurement capability.

Procedural changes during this period included:

- care to exclude sources of hydrogen gas from the gas-bulb environment;
- care to avoid pressure inequalities across the gas-bulb wall, particularly above 327 °C;
- greater use of “integrity checks” to discover the presence of impurity gases or leaks in the gas thermometer;
- use of an improved procedure for evaluation of the dead-space correction;
- use of greater care in handling and evaluating PRTs;
- use of virial coefficients to calculate the non-ideality correction for ^4^He instead of multiple pressure values in the gas thermometer measurements; and •introduction of computers for data analysis.

### 6.1 Fused Salt Bath

In order to attain still higher temperatures in the gas thermometer bulb assembly, Guildner and Edsinger built a stirred liquid bath of the same general type described in section 4, but containing as the bath medium a eutectic mixture of lithium, sodium, and potassium nitrates. The use of this medium for thermostat baths has been described briefly by Beattie [14]. As before, the gas bulb assembly, along with the constant-volume valve block, the thermostated capacitance diaphragm gage, and the gas analysis system, was lifted hydraulically in order to change the environment from an ice/water or water/potassium-chromate bath to the fused salt bath.

The fused salt bath could be used only to 457 °C, owing to increasing tendencies of the liquid nitrate mixture to attack the thermometer sheaths and to migrate from the reservoir into the heater-insulator portion of the bath. Therefore, it was decided to investigate the temperature region from 0 to 457 °C before building a new thermostat for higher temperatures.

### 6.2 Thermal Expansion Measurements

Although the Pt-Rh alloys from which all the NBS/NIST gas bulbs have been fabricated are stable, high-temperature materials, they also exhibit large coefficients of thermal expansion. In order to determine thermodynamic temperatures at a level adequate for use in the NBS/NIST gas thermometry program, it was necessary for Guildner and his colleagues to obtain linear thermal expansion measurements from the gas-bulb materials over the entire range of the gas thermometry measurements with an accuracy approaching 1 ppm. This level of accuracy was not easy to achieve by use of ordinary thermal expansion equipment (even with Fizeau interferometry), because it utilized thermocouple sensors of limited accuracy [16,17].

**Figure 9:**
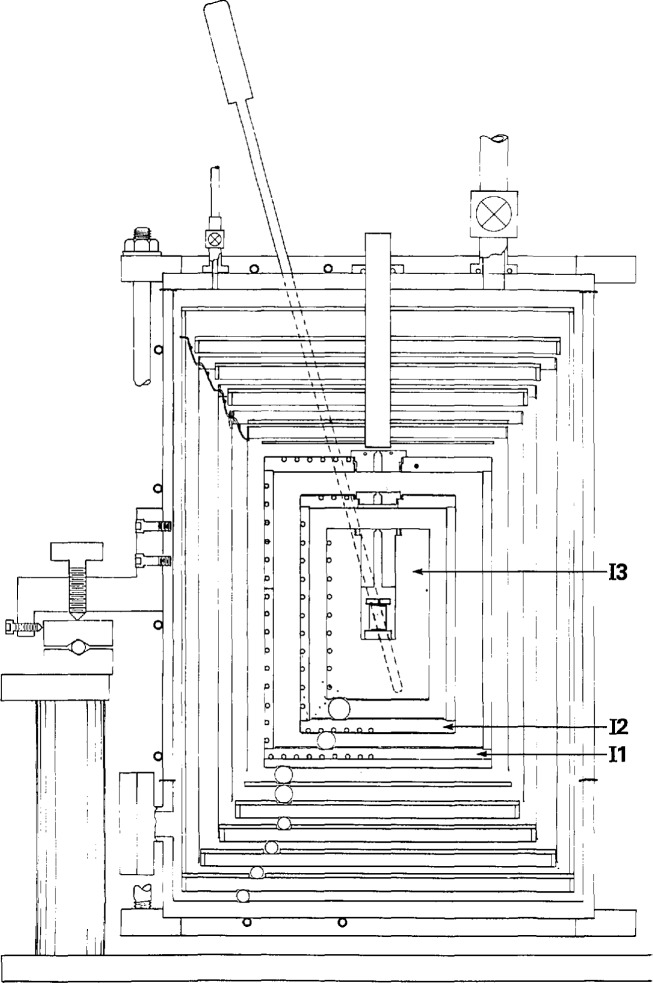
Cross-section drawing of original NBS/NIST gas-thermometry thermal expansion furnace. The outermost shell was made of brass in three sections. PTFE gaskets were employed at the joints to permit evacuation of the furnace. Cooling coils on the outside surface of the outer shell could be connected to refrigerated fluid in order to cool the furnace to temperatures as low as −30 °C. Successively smaller radiation shields of copper, silver (three shields), and gold enclosed three Inconel 600 shields, I1, I2, and I3. Shell I1 was equipped with separate resistive heaters placed in grooves in its top, bottom, upper sidewall, middle sidewall, and lower sidewall. Shell I2 supported heater circuits located on its top plate, on its sidewall, and on its bottom plate. The innermost shell, I3, was prepared from a solid cylinder of Inconel of 7.5-cm diameter and 13-cm length; a central hole was bored to house the sample assembly, and an off-center hole accommodated a calibrated PRT. Three-element “Platinel” thermocouples were arrayed along the surfaces of shells I1, I2, and I3; these thermocouples were connected in differential circuits for temperature control. A quartz window, 1.9-cm diameter and 16-cm thick, was sealed in place above the sample. Sample loading was accomplished by removal of the lids of the shields and the slotted plugs in the Inconel shells. All shields and shells rested upon steel balls that were constrained by slots in the next lower plate. The furnace could be rotated on a ball-bearing assembly; it was leveled by means of three sets of screws that contacted the circular rotation unit as shown in the figure. (Reprinted from reference [19].)

**Figure 10:**
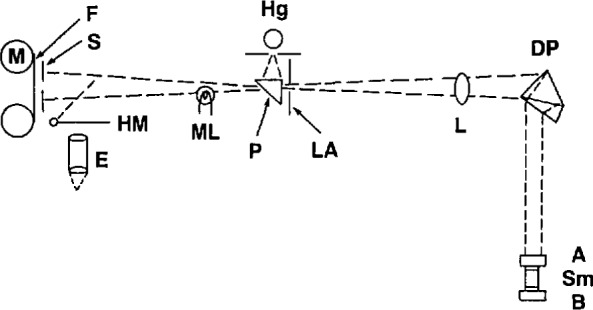
Schematic drawing of the recording interferometer [36,37,38]. A low-pressure electrodeless ^198^Hg lamp, Hg, provides light for the interferometer through a prism, P, a limiting aperture, LA, lens L, and a double dispersing prism, DP. The light is partially reflected by the lower surface of optical plate A and then fully reflected by the upper surface of plate B; the two plates enclose the sample, Sm, and with it create the interference fringe pattern that falls upon the narrow horizontal slit S and is recorded on 35 mm panchromatic film F. A hinged mirror, HM, and eyepiece, E, allow the operator to view the fringe pattern before recording it. A drive mechanism, M, moves the film at one of three pre-chosen rates. A marker lamp, ML, can be used to identify particular locations on the film. (Reprinted from reference [19].)

**Figure 11:**
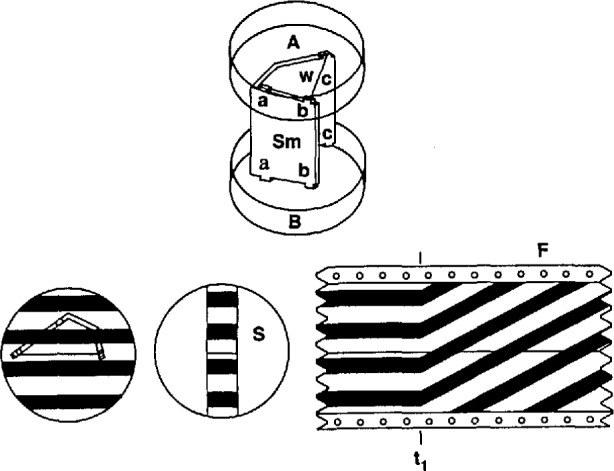
Sample assembly and fringe pattern. The upper surface of the upper optical plate, A, makes an angle of 20° with respect to the lower surface, so that its reflections do not appear in the field of view. The lower surface is coated with TiO_2_ for 30% reflection. The upper surface of the lower optical plate, B, is coated for total reflection, while its lower surface was left rough. The sample, Sm, is shaped so as to provide three-point support for the upper plate. Three sets of tabs, a–a, b–b, and c–c were left on the upper and lower sample edges for fine adjustment of the plate separation, so as to produce a fringe pattern similar to that shown in the lower left corner of the drawing. The pattern is partially blocked by a slot S in the central copper tempering block, providing a filmed image that is fixed with respect to the fiducial wire, w, during steady-temperature recording. At time *t*_1_, the film, F, moving leftward, begins to record the moving fringe pattern resulting from a change in sample temperature. (Reprinted from reference [19].)

During the mid-1960s, a special thermal expansion apparatus was designed and constructed for the accurate determination of the linear thermal expansion of materials from which NBS/NIST gas thermometer bulbs had been prepared. Based upon the principles of Fizeau interferometry and precision thermometry, that apparatus consisted of a sample chamber surrounded by a copper block that was in turn enclosed within some seven concentric thermal shields [18,19]. Figure 9 shows the dilatometer furnace that enclosed the sample, figure 10 shows the interferometer that was employed to observe and register the sample thermal expansion, and figure 11 shows the sample assembly and the fringe pattern as recorded on panchromatic film. Temperature control of the sample was achieved by manual adjustment of some nine heaters located on the three inner shells of the furnace; the settings were arranged to minimize the interior temperature gradients as indicated by sets of differential thermocouples. The sample temperature was measured with an estimated accuracy of 0.01 °C by use of a specially constructed platinum resistance thermometer. Use of a homemade fringe-reading system allowed measurement of the sample thermal expansion with an imprecision of about 0.01 fringe.

Accurate determination of thermal expansion with the apparatus described above was a complicated process. It involved careful preparation of samples so as to obtain a suitable fringe pattern; recording on film the fringe pattern both at a series of stable, carefully measured temperatures and during the intervening temperature changes; regulating and measuring the pressure of the heat-exchange gas at the fixed-temperature points; and determining from the filmed patterns the fringe changes that accompanied each modification of the temperature. The thermal expansion coefficients were obtained by fitting the experimental data by means of equations such as

$$\Delta N\left(t_{i},t_{i+1}\right)=\sum_{n=1}^{m}A_{n}\left(t_{i+1}^{n}-t_{i}^{n}\right).$$ (7)

Detailed discussions of the thermal expansion apparatus, the measurement procedure, and one method of analyzing data obtained during experiments performed some time ago, are given in reference [19]. Here, we shall summarize the results obtained for the percent linear thermal expansion in the range 0 to 550 °C, with reference to 0 °C, for 100% Pt:

$$\begin{array}{l} 100\in \left(t,0{}^{\circ}C\right)=100\left[L\left(t\right)-L\left(0{}^{\circ}C\right)\right]/\left[L\left(0{}^{\circ}C\right)\right]\\ =8.862\times 10^{-4}t+1.760\times 10^{-7}t^{2}\\ -1.444\times 10^{-10}t^{3}+8.93\times 10^{-14}t^{4}. \end{array}$$ (8)

For 88 Wt% Pt+12 Wt% Rh

$$\begin{array}{l} 100\in \left(t,0{}^{\circ}C\right)=8.763\times 10^{-4}t+2.116\times 10^{-7}t^{2}\\ -1.455\times 10^{-10}t^{3}+1.036\times 10^{-13}t^{4}, \end{array}$$ (9)

and for 80 Wt% Pt+20 Wt% Rh

$$\begin{array}{l} 100\in \left(t,0{}^{\circ}C\right)=8.674\times 10^{-4}t+2.538\times 10^{-7}t^{2}\\ -2.801\times 10^{-10}t^{3}+1.480\times 10^{-13}t^{4}. \end{array}$$ (10)

The estimated standard deviation of the fit to the data in each case was less than 0.1 fringe (<1 ppm in sample length).

Because of a slight difference in fitting procedure from that described in reference [19], the equation corresponding to eq (9) that was actually used for the (88/12) alloy in the gas thermometry calculations in the range 0 to 457 °C was as follows:

$$\begin{array}{l} 100\in \left(t,0{}^{\circ}C\right)=8.7509\times 10^{-4}t+2.2782\times 10^{-7}t^{2}\\ -2.4767\times 10^{-10}t^{3}+3.462\times 10^{-13}t^{4}\\ -1.9117\times 10^{-16}t^{5}. \end{array}$$ (11)

At 457 °C, use of the newer coefficients (from reference [19]) would have resulted in a higher calculated value of the Kelvin Thermodynamic temperature (see next section) by 0.003 °C.

## 7. Scale Differences From 0 to 457 °C

The characteristics of the second version of the NBS/NIST gas thermometer indicated the capability for determination of Kelvin thermodynamic temperatures up to 457 °C with very low uncertainty levels, although it still was necessary to record data by hand and to make use of the central NBS facility for the computation of results.

A comprehensive presentation of results from the NBS gas thermometer was published in 1976 by Guildner and Edsinger [21]. The work described in that paper embodied the changes from earlier efforts in both equipment and procedures that are mentioned briefly above. The earlier results were slightly modified, and they were substantially extended in range.

Measurements that were part of each determination of a Kelvin thermodynamic temperature with the revised apparatus included the following:

- Checks on the integrity of the capacitance diaphragm gage and of the capacitance bridge used in setting the manometer mercury levels, to ascertain that these vital systems were working properly;
- Resistances of three long-stem platinum resistance thermometers inserted into the gas-bulb protective case, followed later by measurements of the thermometers in a triple point of water cell, to provide an estimate of the gas-bulb temperature on the IPTS-68;
- Voltages of more than ten thermocouples that were ranged along the gas-bulb capillary, referred to ice junctions, to provide part of the dead-space correction;
- Resistance of a long-stem PRT placed on the manometer block, and voltages of a set of thermocouples ranged along the manometer mercury lines, to provide the temperature of the mercury in the manometer;
- Height of the stack of wrung gage-block end standards under the pumped cell of the manometer, to provide the height of the mercury column;
- Capacitance of the diaphragm gage that separated the manometer gas from the gas thermometer working gas, to provide a value of the pressure difference.

Most of the types of measurements made by Guildner and Edsinger became part of the procedures used in later measurements to 660 °C. Therefore it is useful to summarize the approach used in the measurements to 457 °C.

### 7.1 IPTS-68 Temperatures

Over the period during which the measurements to 457 °C were performed, four calibrations of the three PRTs used in the gas bulb and the PRT used to measure the temperature of the manometer were obtained. The fixed-point resistance values repeated at the level of 0.00045 °C. The constants obtained in the calibrations were employed in a Fortran IV program that was run as needed on the NBS computer in order to provide the mercury temperature and the gas bulb temperature corresponding to each pressure-temperature determination. The symbolic program was given in Appendix I of reference [21]. It was estimated that the uncertainty in the calculated IPTS-68 temperatures never exceeded 0.001 °C.

IPTS-68 temperatures of copper-constantan thermocouple thermometers were calculated from a standard table of emf vs. temperature [22]. IPTS-68 temperatures of Pt vs. (Pt+10 Wt% Rh) thermocouple thermometers were obtained by use of an equation derived from calibrations of thermocouples similar to those employed in the gas thermometer:

$$\begin{array}{l} E=5.45846\times 10^{-3}t+1.13497\times 10^{-5}t^{2}\\ -1.52447\times 10^{-8}t^{3}+9.06033\times 10^{-12}t^{4}. \end{array}$$ (12)

The above equation represented the observed data within 0.47 *μ*V from 25 up to 500 °C.

### 7.2 Manometer Pressure

Calculation of the pressure on the manometer side of the capacitance diaphragm gage is discussed at length in reference [5]. Each pressure value had several components:

1. the height of the gage blocks stacked below the upper cell of the manometer, modified by thermal expansion (calculated from the difference between the calibration temperature and the measured manometer temperature) and by the compression resulting from the weight of the upper cell;
2. the density of the mercury, obtained from the following relation: 1.354584×10^−4^ [1–1.8110 × 10^−4^ (*t*−20 °C)+3.8× 10^−11^ *ρ* (in N/m^2^)] kg/m^3^ [40,41,42]. The temperature was obtained in each case by reading the manometer PRT. The pressure, arising from the head of mercury, was calculated for one-half the gage-block stack height;
3. the acceleration due to gravity, *g*, 9.801022 [1–3×10^−7^ *h* (in m)] m/s^2^ [43], where *h* was the vertical height of the capacitance gage above the reference level at which the value of g was originally determined;
4. the hydrostatic head of ^4^He gas between the manometer and the diaphragm, *π*_m_, given by
$$\pi_{m}=\sum_{k}\left[M\;g\;l_{k}\right]/\left[R\;T_{k}\right],$$ (13)
  where M denotes the molecular weight of the gas, *l_k_* stands for an increment of length, *R*, the molar gas constant, is 8.3137×10^6^ [cm^3^ Pa]/[mol K], *T_k_* represents the temperature of the *k*th element of length of the riser tube that connected the two instruments, and the summation occurs over the full length of the riser tube; and
5. any difference in the partial pressure of mercury on the two sides of the manometer. One notes in this component of the pressure calculation that the use of a cooled mercury vapor diffusion pump to maintain a vacuum over the upper cell may result in a lower partial pressure of mercury over the upper cell than that existing in the riser tube above the lower cells.

### 7.3 Gas Bulb Pressure

To convert the pressure evaluated at the manometer side of the diaphragm to the value existing within the gas bulb, it was necessary to calculate or measure the pressure difference across the diaphragm, the pressure head between the diaphragm and the gas bulb, and the thermomolecular pressure correction for the capillary.

The sensitivity of the diaphragm gage was periodically re-determined by observing the values of capacitance that corresponded to small, measured gas pressures introduced into one side of the gage. It was necessary as well to maintain a frequent schedule of null-pressure measurements to verify the capacitance value that corresponded to equal pressures on the two sides of the diaphragm.

The pressure head between the diaphragm gage and the center of the gas bulb was obtained from a relation similar to eq (13), with the additional consideration of the thermal expansion of each element of the capillary:

$$\pi_{c}=[\left\{M_{g}\right\}/\left\{RK\right\}]\sum_{k}\left[l_{k}/T_{k}\right]\left[1+\alpha_{k}\Delta t_{k}\right].$$ (14)

In eq (14), the *α_k_* and Δ*t_k_* represent, respectively, the linear thermal expansion, and the difference from 23 °C of the temperature, of each element. The summation runs over the length of the capillary from the diaphragm to the center of the gas bulb. Other symbols have the meanings given in eq (13).

The thermomolecular pressure correction appropriate for each measurement was estimated on the basis of experiments performed with a capillary similar to the one used in the gas thermometer. The substitute capillary was enclosed within a larger tube (about 9.6 mm i.d.). In the experiments, the capillary was connected to one side of the diaphragm gage and the larger tube to the other; the assembly was installed in a vacant gas-bulb thermometer well (see fig. 2 of reference [21]). By determining the pressure difference between the two tubes as functions of pressure and temperature, Guildner and Edsinger obtained values for the thermomolecular pressure corrections up to 457 °C that they estimated to be accurate within about 1%.

### 7.4 Gas Bulb Volume

The absolute interior volume of the gas bulb at the reference temperature needed only be known with moderate accuracy; a change of 8% in the evaluation of the bulb volume was estimated to effect only a 0.003 °C modification in the calculated thermodynamic temperature near 150 °C. Measurement of the volume better than this level of accuracy was readily accomplished by weighing the bulb empty and then weighing it filled with water at a known temperature.

Much more significant was any uncertainty in the thermal expansion of the gas bulb from the reference temperature to the test temperature. The volume of a gas bulb at any temperature *t*_u_, *V*(*t*_u_), could readily be calculated once the linear thermal expansion of the gas-bulb material had been determined for the temperature range of interest:

$$\begin{array}{l} V\left(t_{u}\right)=V\left(t_{r}\right)\left[1+\beta \left(t_{u}-t_{r}\right)\right]\\ =V\left(t_{r}\right){\left[1+\bar{\alpha }\left(t_{u}-t_{r}\right)\right]}^{3}, \end{array}$$ (15)

where $\bar{\alpha }\left(t_{u}-t_{r}\right)=\left[1\left(t_{u}\right)-1\left(t_{r}\right)\right]/\left[1\left(t_{r}\right)\right]\left[t_{u}-t_{r}\right]$.

In the case of the experiments reported in reference [21], a complication arose because the gas bulb top had been fabricated from 80 Wt% Pt/20 Wt% Rh alloy, whereas the sides and bottom had been made from 88 Wt% Pt/12 Wt% Rh alloy. Because of the slight difference in thermal expansion coefficients, the shape of the cylindrical gas bulb could be expected to change with temperature. Considering the probable nature of the distortion, Guildner and Edsinger found that the change in volume of the bulb could be adequately calculated by using the mean thermal expansion coefficients characteristic of the 88/12 alloy.

### 7.5 Dead Space Evaluation

We noted in section 4 that the procedures used during gas-thermometer measurements restricted the dead space to the gas-bulb capillary and the constant-volume valve that closed it off from the capacitance diaphragm gage. We also mentioned there that the uncertainty contributed by the constant-volume valve was negligible. The temperature correction resulting from the amount of the working gas that was contained in the capillary could be calculated on the basis of eq (4). Dividing the total number of moles of working gas in the gas thermometer between the gas bulb and the dead space (essentially all of which consisted of the capillary) and neglecting, for this purpose, the non-ideality of the gas, we could evaluate the temperature correction arising from the dead space, *δt*_ds_, as

$$\begin{array}{l} \delta t_{\mathrm{ds}}=T_{\mathrm{bu}}\left(\text{including dead space}\right)\\ -T_{\mathrm{bu}}\left(\text{no dead space}\right), \end{array}$$ (16)

where the two right-hand terms refer to the bulb temperatures calculated for the higher-temperature state first including the capillary and then neglecting the capillary. The first term can be written

$$T_{\text{bu}}=\left[P_{\text{bu}}V_{\text{bu}}\right]/[\left\{P_{\text{br}}V_{\text{br}}/T_{\text{br}}\right\}+\sum_{k}\left(P_{\text{kr}}V_{\text{kr}}/T_{kr}\right)-\sum_{k}(P_{\text{ku}}V_{\text{ku}}/T_{ku}],$$ (17)

where all volumes *V* are corrected for thermal expansion and the capillary pressures *P_k_* are corrected for hydrostatic head and thermomolecular effects. The second term on the right-hand side of eq (16), in which the dead space correction is neglected, is simpler:

$$T_{\text{bu}}=\left[T_{\text{br}}P_{\text{bu}}V_{\text{bu}}\right]/\left[P_{\text{br}}V_{\text{br}}\right].$$ (18)

With only a negligible error, eq (16) can be re-written in the form

$$\delta t_{\text{ds}}=-[\left\{T_{\text{br}}T_{\text{bu}}\right\}/V_{\text{br}}]\sum_{k}[\left\{V_{\text{kr}}/T_{\text{kr}}\right\}-\left\{P_{\text{bu}}V_{\text{ku}}\right\}/\left\{P_{\text{br}}T_{\text{ku}}\right\}].$$ (19)

The temperatures of the individual elements of the capillary were deduced from measurements of the thermocouples arrayed along its length.

### 7.6 Effect of Gas Non-Ideality

The method used in the earliest series of gas thermometry measurements to eliminate errors arising from the non-ideality of the ^4^He working gas was to perform a set of measurements at the same unknown temperature with several filling pressures; extrapolation of the indicated gas thermometer temperature to the value corresponding to zero filling pressure could then compensate for the non-ideality of the gas. However, the magnitude of the thermomolecular correction at low pressures and high temperatures increased the uncertainty from that source, so that, in the work described in reference [21], the virial theorem was employed, with values of the second virial coefficient obtained from the existing literature.

An equation was developed to represent the second virial coefficients of ^4^He over the range 0 to 600 °C:

$$B\left({\mathrm{cm}}^{3}/\text{mol}\right)=11.9967-4.48574\times 10^{-3}t+1.46724\times 10^{-6}t^{2}.$$ (20)

The estimated uncertainties in the non-ideality corrections resulted in uncertainties in the KITS temperatures that ranged from 0.12 mK at 100 °C and 20 kPa to 1.23 mK at 457 °C and 100 kPa (reference [21], sec. 9).

### 7.7 Summary of Results

As a result of a series of 123 sets of measurements with the gas thermometer over a period of 15 months, Guildner and Edsinger derived a relation (rounded off here to five significant figures) for the difference between the Kelvin Thermodynamic Temperature Scale and the IPTS-68 in the range 0 to 457 °C (273.15 to 730.15 K):

$$T/K-T_{68}/K_{68}=-1.2089\times 10^{5}/{T_{68}}^{2}+1.2135\times 10^{3}/T_{68}-4.3160+6.4408\times 10^{-3}T_{68}-3.5664\times 10^{-6}{T_{68}}^{2}.$$ (21)

The uncertainties associated with the major components of the measurements contributing to these results are summarized in table 2.

**Figure 12:**
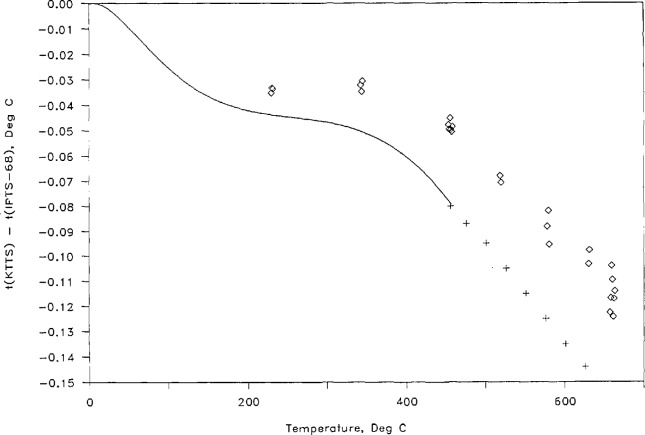
Temperature scale differences *t*(KTTS)-*t*(IPTS-68) as determined with various versions of the NBS/NIST gas thermometer. Solid line—results given in reference [21] by Guildner and Edsinger. Diamonds—results given in reference [35] by Edsinger and Schooley [35]. For comparison with the higher-temperature results of reference [35], the results (indicated by plus signs) obtained by Jung [25], using radiation thermometry based on the results of reference [21] at 457 °C, are included as well. (Reprinted from reference [35].)

The differences *t*(KTTS)-*t*(IPTS-68) at the steam point, the tin point, the zinc point, and 457 °C (the highest temperature reached), along with their estimated uncertainties, are given in table 3. The results are shown in graphical form in figure 12.

Inasmuch as the NBS gas thermometry results showed deviations of the IPTS-68 from its thermodynamic counterpart that were everywhere at least twice as large as had been estimated during its formulation in 1968, considerable effort has been expended in other laboratories to seek corroboration of the gas thermometer determinations by different experiments. Recently, similar results below 140 °C have been found using the methods of total radiation thermometry [23] and acoustic thermometry [24].

## 8. Most Recent Version of the NBS/NIST Gas Thermometer

After Guildner and Edsinger reported on the deviation of the International Practical Temperature Scale of 1968 from the Kelvin Thermodynamic Scale in the range 0-457 °C, Jung [25], Coates and Andrews [26], and Coates, Andrews, and Chattle [27], performed spectral radiation thermometry experiments in order to realize the KTTS at still higher temperatures. To obtain measurements that were consistent with the NBS gas thermometry. they each utilized the NBS determinations near 457 °C as reference temperatures.

Because of the difficulty involved in performing spectral radiation thermometry at temperatures as low as 457 °C, experimental uncertainty at that temperature is considerably larger than it is at higher temperatures; furthermore, systematic errors in “low-temperature” radiation thermometry become increasingly difficult to evaluate. Since in principle gas thermometry can provide reference temperatures substantially above 457 °C that are referred to the principal KTTS defining temperature at 0.01 °C, it was desirable to continue study of the KTTS by gas thermometry at temperatures above 457 °C. A further advantage to be gained by such a step is that an extended range of the KTTS would be explored by two methods that possess intrinsically different systematic errors, allowing better evaluation of the uncertainty of thermodynamic temperatures throughout the overlapping range. For these reasons, R. E. Edsinger and the present author recently undertook experiments that continued the work of Guildner and Edsinger to up temperatures as high as 660 °C.

Some of the apparatus built at the NBS for gas thermometric studies above 500 °C was described by Guildner and Edsinger in 1982 [28]. It was necessary to modify that equipment in the course of using it to determine the difference *t*(KTTS)-*t*(IPTS-68) from 230 to 660 °C in the most recent experiments. It also proved desirable to modify the furnace used for the determination of thermal expansion; the gas bulb; the gas-bulb thermometers; the method used in purifying the “He working gas; and the technique for tracking the “counterpressure.” No changes were required in the manometer.

### 8.1 High-Temperature Furnace

The major difference between the most recent gas therraometry experiments and those reported previously involved the thermal environment of the gas bulb. In the earlier experiments up to 457 °C, the gas-bulb/case system was immersed, as needed, in liquid baths; in an ice/water bath to provide the reference state, in a water/potassium-chromate bath to provide uniform temperatures between 0 and 140 °C, or in a bath containing a eutectic mixture of lithium, sodium and potassium nitrates to provide uniform temperatures between 140 and 457 °C. As noted in section 6, use of the molten salt bath above 457 °C, while providing quite uniform temperatures in the volume containing the gas bulb, nevertheless offered its own problems; not only did the hot molten salts attack the sheaths of the PRTs, but the solution also migrated out of the bath reservoir, occasionally damaging the electrical circuits.

**Figure 13:**
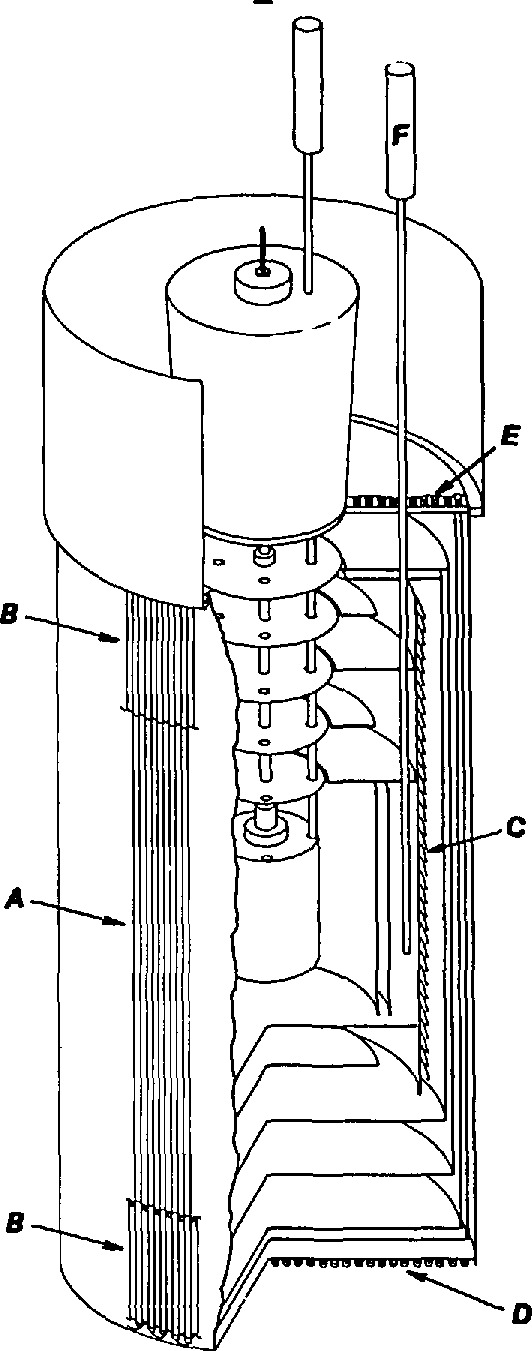
Cutaway drawing of the inner, heated portion of the high-temperature gas thermometer thermostat, shown with the gas thermometer assembly lowered into its measurement position. Like the other NBS/NIST Gas Thennometry thennostats, the 200–1000 °C furnace was movable. The exterior surface of the thermostat (not shown here) was cooled by chilled water flowing through coils brazed to it. The heat load on the exterior surface was reduced by the inclusion of 20 cm of Fiber-frax insulation between it and the heated portion shown here. The furnace was gas-tight except at the top. Argon gas was inserted through the exterior wall into the insulated space outside the heaters A,B and also into the volume surrounding the protective case of the gas bulb, in order to reduce the concentration of hydrogen and other active species in the thermostat The inner parts of the furnace were made of Inconel alloys. The shields inside the heated portion of the thermostat provided isolation from the laboratory environment and support for some eight independent zone heaters. The main heaters. A, were composed of vertical windings of Nichrome wire threaded through single-hole ceramic insulators. The heater was divided into independently controlled quadrants to allow for slight misalignment of the gas-bulb assembly with respect to the furnace centerline. Band heaters, B, consisting of short sections similar to the main heaters, surrounded the top and bottom of the main heaters. A regulating heater, C, distributed its heat evenly over one of the shields. Its power was regulated by the temperature as sensed by a control thermometer, F. A bottom heater, D, a top heater, E, and three heaters in the gas-bulb suspension assembly could be adjusted independently to correct vertical temperature gradients. Approximately 750 W of overall heater power were required to maintain the gas bulb at 660 °C; less than 1% of the total was dissipated in heater C. A line conditioner was used to regulate the line voltage to the heater circuits, and specially designed controls were used on the heaters. A soft fiberglass rope was used to seal the conical plug on the gas-bulb suspension into its socket prior to each run. (Reprinted from reference [28].)

A less homogeneous thermal environment was provided for the most recent experiments by an argon-filled furnace [28]. A schematic drawing of the high-temperature furnace is shown in figure 13. Although capable of providing gas-bulb temperatures as high as 1000 °C, some eight heaters required careful manual adjustment if the operator wished to eliminate very noticeable temperature gradients in the gas bulb. The furnace response time was an hour or more, depending upon its temperature; however, its thermal stability was quite high, permitting ample time for measurements after adjustment. As before, an ice bath was used to provide the reference state near 0 °C for the gas bulb.

### 8.2 Gas Bulb Assembly

The gas bulb in use during the most recent experiments was not the one that was used in the measurements reported earlier [21], although it was quite similar in its construction (see fig. 7). The major change in its construction was in its lower end cap, which was more than doubled in thickness for reasons to be discussed later. The gas bulb was made entirely of 80 Wt% Pt + 20 Wt% Rh in the shape of a right circular cylinder of volume ~407 ml. As before, it was connected to a capacitance diaphragm gage via a capillary of composition 90 Wt% Pt + 10 Wt% Rh. Because the thermostat provided a relatively poor level of temperature homogeneity in comparison to the liqviid baths used in earlier versions of the gas thermometer, more capillary thermocouple stations were added to define the temperature profile along the capillary immediately above the gas bulb. The capillary/gas-bulb system was enclosed by the same heavy-walled Inconel 600 protective case and tube that had been used in the previous experiments. Helium gas again was introduced into the protective case and tube at nearly the same pressure (the “counterpressure”) as that existing at any given time in the gas bulb [20].

Invariably, four platinum resistance thermometers (PRTs) were placed in the four wells in the wall of the protective case prior to measurements; these were used to determine IPTS-68 temperatures of the gas bulb. Radial temperature gradients within the gas bulb could be evaluated by comparing the temperatures of the PRTs at a fixed depth in the case, and minimized by adjusting separately the thermostat heaters (A, fig. 13). Vertical gradients could be detected by varying the vertical positions of the PRTs.

As before, the operating procedures were chosen so that the dead space correction applied only to the capillary above the gas bulb and to the constant-volume valve that was located directly above the capillary. The magnitude of the dead-space correction was now determined by measuring the emf of each of some 22 (Pt) vs (Pt + 10 Wt% Rh) thermocouples that were arrayed along the capillary between the gas bulb and the constant-volume valve.

### 8.3 Platinum Resistance Thermometry

A set of calibrated high-temperature PRTs was used to control the furnace, to monitor the temperature uniformity in the gas bulb, and to provide its IPTS-68 temperature. The thermometers were especially designed for use at temperatures as high as 1000 °C; they were made with 2.5 Ω nominal ice-point resistance, bifilar helical windings, and synthetic vitreous silica sheaths. Most of these thermometers were made at the NBS, either by J. P. Evans [28,29,30] or by Guildner and Edsinger [28]; during the last stages of the experiments, however, two more were obtained from a commercial source [45].

Two resistance bridges of NBS construction were used to measure the resistances of the PRTs. One of these bridges, a 400-Hz, manually balanced model [31], monitored a PRT that was located near furnace heater C (see fig. 13); it was used for fine temperature control. The heater supply derived its signal from the bridge output. A second resistance bridge of more recent design [32] provided an automatic-balancing, computer-controlled monitor for the four PRTs that were used in the protective case of the gas-bulb system, as well as for a standard PRT that continually monitored the manometer cellar temperature.

### 8.4 Auxiliary Equipment

Purified ^4^He gas was obtained from the same high-pressure cylinder that was used in the work published earlier. Before it was allowed to enter the gas bulb or manometer, the helium was passed slowly (~0.06 mol/h) through a copper trap that was surrounded by liquid ^4^He at 4.2 K. This purification procedure is quite different from the one used previously (see sees. 4 and 5). Some of the helium gas was diverted to a 3-L storage volume so that small volumes of the system (typically, the constant-volume valves and the capacitance diaphragm gage) could be emptied and refilled during a measurement sequence.

A digital capacitance diaphragm gage was introduced to monitor the pressure in the manometer-counterpressure system while it was being filled or emptied, and during furnace temperature changes [44]. (At these times, the mercury system in the manometer was valved off to prevent flooding of the cells and consequent shorting of the level-sensing capacitance plates.)

A low-thermal, four-deck scanner was used in conjunction with a digital voltmeter and a laboratory microcomputer to monitor a variety of measurements. These included the following:

- Resistance of the thermometer-bridge resistance standard contained in a thermostated enclosure;
- Resistances of the four PRTs in the gas-bulb protective case;
- Resistance of the PRT in the manometer cellar;
- EMFs of the 22 [Pt vs. (Pt + 10 Wt% Rh)] thermocouple thermometers located along the gas-bulb capillary, each measured against a reference junction held at the ice point, and emfs of the four thermopiles located on the mercury lines and cells of the manometer;
- Output of the capacitance diaphragm gage that monitored the counterpressure;
- EMF of the thermocouple thermometer that monitored the temperature of the main capacitance diaphragm gage; and
- Voltages appearing on the manually set high-temperature furnace heaters.

Calibration data recorded in the memory of the microcomputer allowed expression of all thermometer readings directly in °C and of the counterpressure capacitance diaphragm gage out-put in pressure units.

### 8.5 Recent Thermal Expansion Measurements

In section 6.2. we described the first of two devices for the precise measurement of thermal expansion, each comprised of a Fizeau interferometer, a thermally homogeneous sample furnace, and a calibrated PRT. The first apparatus was used to measure the thermal expansion of the materials from which the gas bulbs used in the earlier gas thermometer experiments were constructed [19,21,28]. The results obtained with this apparatus were of high quality, with an imprecision generally better than 1 ppm in sample length; however, setting the sample temperature to a stable, uniform value required the iterative adjustment of the current settings in nine furnace heaters. This technique proved to be both time-consuming and tedious. The advent of heat-pipe thermal equilibration techniques led to the re-building of the thermal expansion furnace, employing a heat pipe in place of the multiple shields.

The objective in modifying the earlier furnace was to simplify its operation while still retaining a satisfactory level of accuracy in the determination of thermal expansion. To reduce the uncertainty of the thermodynamic temperatures arising from uncertain knowledge of the thermal expansion of the gas bulb to the level of ±0.011 °C at 660 °C (approximately 12 ppm of the temperature in kelvins), it was necessary to measure the thermal expansion of the gas bulb material accurately within about 4 ppm in sample length. This objective appeared to be achieved with the construction of a simpler, computer-controlled furnace, A recent paper [33] contains a description of the construction of the new furnace and its use to determine the coefficients of linear thermal expansion of Pt and Pt-Rh alloys in the range −20 to +700 °C.

**Figure 14:**
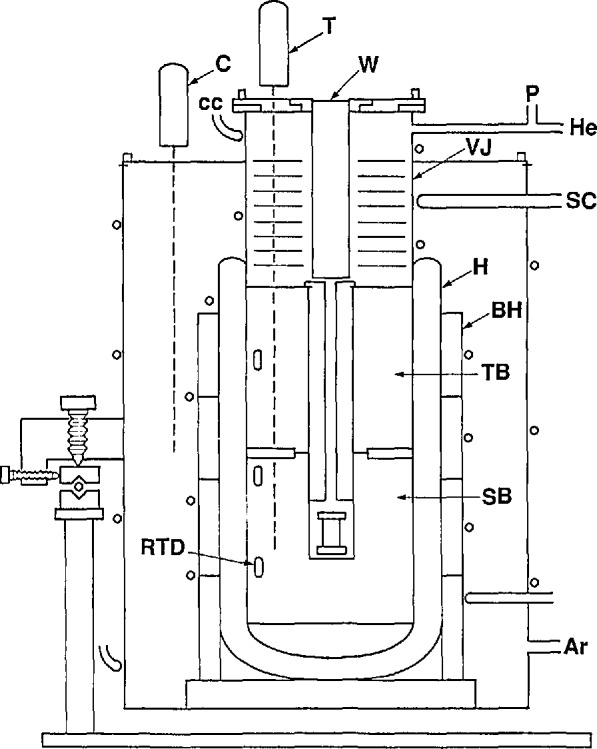
Cross-section drawing of heat-pipe thermal expansion furnace. The outer wall was made of brass; a cooling coil, cc, made of copper tubing was brazed to the outside. A slight overpressure of argon gas was maintained inside the outer wall through the connection Ar. The furnace was supported at three points on a turntable that rested upon the laboratory bench. A closed-end, Inconel heat pipe, H, loaded with 50 g of potassium metal, rested upon an insulating support. Four ganged band heaters, BH, provided heat to the heat pipe. A platinum resistance thermometer, C, sensed the temperature in the vicinity of the band heaters, allowing rough temperature control. A sealed tube, VJ, served as an evacuable enclosure for the sample assembly. The sample itself was centered within the lower of two copper blocks, SB, which also provided a socket to receive a calibrated platinum resistance thermometer, T. The upper copper block, TB, tempered the ^4^He fill gas, thermometer T, and a central slotted copper block. A quartz window, W, tempered by a set of Inconel baffles, admitted light from the interferometer. A coil of stainless steel tubing, SO, was used to cool the sample chamber below room temperature. Ports P and He were used to monitor the fill-gas pressure and to evacuate the sample space, respectively. (Reprinted from reference [33].)

Figure 14 shows the heat-pipe furnace. A sealed outer wall was constructed in two parts that were screwed together onto a PTFE gasket. A six-turn copper cooling coil was brazed onto the outer wall of the furnace. Argon gas was fed into the furnace at a pressure slightly above ambient so as to reduce the likelihood of water or other unwanted impurity gases inside the furnace.

An Inconel heat pipe (labelled H in fig. 14) containing 50 g of potassium metal was obtained commercially. The lower end of the heat pipe was closed, providing a Dewar-like configuration. A close-fitting Inconel jacket, VJ, inside the heat pipe, provided a vacuum-tight sample chamber. The vacuum jacket permitted the sample chamber to be pumped and filled with gas of any desired composition and pressure. The heat pipe was surrounded by a set of four, 1250-W, cylindrical band heaters, BH, connected in electrical series. A long-stem platinum resistance thermometer, C, was mounted with its sensor near the band heaters to allow rough control of the heat pipe temperature.

Within the space tempered by the heat pipe were placed two copper blocks that were joined lightly as shown in figure 14. The lower copper block, SB, provided a central chamber for the sample assembly and a socket for a second long-stem PRT, T. This thermometer was monitored to obtain sample temperature values. Also included in block SB were two of three small, industrial-grade platinum resistance thermometers (RTD) to be used in evaluating the quality of temperature homogeneity throughout the sample chamber. The upper copper block, TB, of which the main function was that of tempering the PRT and the sample gas, contained the third RTD unit. On the outside of each copper block was wound a resistance heater. The two block heaters were connected electrically in such a way that the proportion of heater power deposited in each could be varied by the operator.

Outside the heat-pipe assembly, a coil of stainless steel tubing, SC, provided the means of introducing a refrigerant into the furnace to allow measurements below room temperature.

The amount of light that impinged upon the sample was restricted by a slotted copper plug above it, by a thick (15 cm) quartz window, W, and by a green filter in the Fizeau interferometer. Since no measurement of the light intensity was performed in these experiments, one can only estimate the level of stray heat from this source.

Potassium was selected as the working substance in the heat pipe despite the fact that its vapor pressure becomes substantial only above 400 °C. Given the relatively low radiative-to-conductive heat transfer properties of the components of the furnace up to that temperature, it seemed likely that the heat pipe would serve as a Dewar vessel up to temperatures at which it became active as a thermal element. The ease of control of the furnace and its thermal stability in use over the experimental range of temperature were consistent with this interpretation.

**Figure 15:**
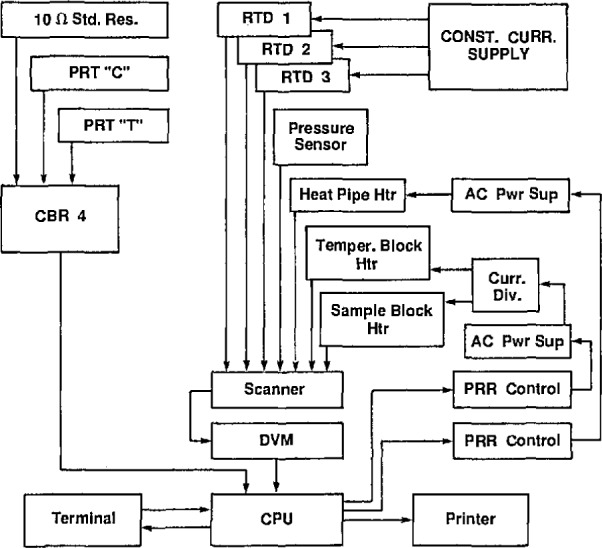
Schematic drawing showing the use of a laboratory microcomputer in acquiring resistance and voltage data and providing control information for dilatometry. The central processing unit, CPU, was programmed to obtain resistance data, using digital bridge CBR4 [32], from a 10-Ω thermostated standard resistor; from a control thermometer, C; and from the sample thermometer, T. The CPU converted those data to temperature values for use in control and evaluation. Through a low-thermal scanner, a digital voltmeter, DVM, provided data from three temperature sensors (RTDs), from a digital pressure sensor, and from various heaters. A portion of the operational program was used to send digital signals to temperature controllers of the proportional-rate-reset type; these, in turn, operated ac power supplies that activated heaters either directly or through current-divider circuits. The CPU also provided out-puts to a terminal and to a printer.

The interferometer, sample assembly, and fringe recording methods were unchanged from the earlier version of the thermal expansion apparatus. However, apart from the recording of the interference fringes, the acquisition of most of the experimental data was accomplished by the use of a dedicated laboratory microcomputer that was programmed to accept digital information from a high-accuracy resistance bridge of NIST construction [32] and from a digital voltmeter. Each of these instruments in turn was connected to several sensors, either directly or by means of low-thermalemf scanners. The microcomputer was used also to activate digital-to-analog power supplies to energize the three furnace heater assemblies, to convert resistance values to temperatures, and to convert digital pressure data from voltage units to pressure units. The pressures thus calculated were further analyzed to yield values of the index of refraction of the sample-chamber gas. Figure 15 is a schematic drawing showing the interconnected components.

The sample temperature was reduced to values as low as −20 °C by the manually regulated flow of nitrogen gas that had been cooled in a heat exchanger held at −195 °C in a bath of liquid nitrogen. As mentioned above, temperatures from ambient to 700 °C were attained by regulating the power to band heaters on the heat pipe assembly and to resistive heaters on the upper and lower copper blocks inside the heat pipe.

The temperature control technique was based upon the use of a dedicated laboratory microcomputer as shown in figure 15. The computer program allowed one to input target temperatures both for the PRT located near the heat pipe (C in fig. 14) and for PRT *T*, used to determine the sample temperature. An input time interval governed the frequency with which the resistances of those thermometers were measured, the corresponding temperatures calculated, and the information displayed on the terminal and, if desired, printed on the printer. The program compared the target temperatures with those determined by measurement, and called for more or less voltage to the heaters depending upon the differences. Limits to the heater voltages could be set by hand, and generally the heat-pipe target temperature was set so as to minimize the power to the tempering block and the sample block. The temperatures calculated for the RTDs were helpful in evaluating the uniformity of the temperature in the two copper blocks. However, the RTDs were not sufficiently stable to be used as the basis for setting the sample temperatures.

In general, use of these techniques allowed the achievement of stable sample temperatures with less than 30 W of power to the tempering block and 4–10 W to the sample block; in contrast, power to the heat pipe heaters ranged from 10 W at 100 °C to about 250 W at 700 °C.

The sample temperature could be resolved within approximately ±0.001 °C in the following manner. A specially prepared PRT was calibrated according to the IPTS-68 and installed in the sample block. The digital resistance bridge permitted the measurement of the resistance of the PRT with a resolution of 1 micro-ohm on command from the dedicated laboratory microcomputer as shown in figure 15. Use of a thermostated resistance standard provided evidence that the bridge measurements were accurate within about 1 ppm. During the times when the furnace temperature was stabilized as described in the previous section, the PRT resistance was read three times, the readings averaged, and the temperature calculated on the basis of the calibration coefficients stored in memory. This process was repeated until the sample temperature appeared to be stable. Then the RTD resistances, the heater voltages, and the sample-chamber pressure were recorded. PRT *T* was then monitored again and the temperature was changed at a pre-determined rate towards the next target temperature.

It may be useful to present here a synopsis of the analytical methods used to obtain values of the thermal expansion as well as certain results that have not been published heretofore. The following expression was used to represent the length of the sample at any Celsius temperature *t_i_*:

$$L(t_{i})=L(t{}^{\circ}C)+\sum_{n=1}^{m}A_{n}[t_{i}^{n}-t^{n}].$$ (22)

In practice, the length of each sample before installation in the dilatometer was measured accurately within about 5 ppm as described in section 6.2. The sample length in fringes of light of frequency 546.2271 nm in vacuum [39] could be expressed by use of the relation

$$N=2L/\lambda_{0},$$ (23)

where *N* denotes the sample length in fringes of light of wavelength *λ*_0_ cm and *L* represents the length of the sample expressed in cm. The values of *N* thus obtained corresponded to the sample length at the bench-top temperature at which the precision micrometer and the gage blocks were employed; later, these values could be corrected to t = 0 °C by use of the fitting equations.

The observed fringe count at the steady-temperature points was corrected for the index of refraction of ^4^He gas, included as a tempering medium in the sample chamber. The approximate 1-atm, 20 °C handbook value, 1.000 036, was corrected to the experimental pressure and temperature by the relation

$$n_{i}=1+0.00003[p_{i}/101][(293)/(t_{i}+273)],$$ (24)

where *p_i_* is expressed in kPa and *t_i_* in degrees Celsius. The correction to the fractional fringe value from the measured to vacuum conditions was then obtained from the relation

$$\Delta N{\left(t_{i}\right)}_{\text{vac}-\text{meas}}=2L_{i}(1-n_{i})/\lambda_{0},$$ (25)

where *L_i_* indicates the sample length at temperature *t_i_.*

To obtain the coefficients of eq (22), the function [47]

$$\Delta N_{\text{vac}}(t_{i},t_{i+1})=\sum_{n=1}^{m}A_{n}[{t^{n}}_{i+1}-{t^{n}}_{i}]$$ (26)

was fitted to the corrected fringe-temperature data sets. This is substantially the same equation that appears earlier in this paper as eq (7). The fitting procedure employed a least-squares computational program with *n* ⩽ 6 coefficients. The program pro-vided an estimated standard deviation of the fit and a coefficient for the evaluation of the significance of each added term in the fitting equation [34].

Evaluation of the coefficients *A_n_* yielded an expression for the linear thermal expansion in the usual form [19]

$$\begin{array}{l} \epsilon (t,0{}^{\circ}C)=[L(t)-L(0{}^{\circ}C)]/L(0{}^{\circ}C)\\ \;=[N(t)-N(0{}^{\circ}C)]/N(0{}^{\circ}C)\\ \;=\sum_{n=1}^{m}\left[A_{n}/N(0{}^{\circ}C)\right]t^{n}. \end{array}$$ (27)

The thermal expansion can be obtained for any other reference temperature from the relation

$$\epsilon (t,t_{\text{ref}})=[\epsilon (t,0{}^{\circ}C)-\epsilon (t_{\text{ref}},0{}^{\circ}C)]/[1+\epsilon (t_{\text{ref}},0{}^{\circ}C)].$$ (28)

Two thermal expansion samples were prepared from the (80 Wt% Pt + 20 Wt% Rh) alloy gas thermometer bulb used in the most recent NIST experiments, using the methods described in section 6.2. We present here details of measurements taken on these samples, labelled S-M and S-U, because they have not been reported heretofore. In addition, they show the level of precision of the thermal expansion measurements obtained using the thermal expansion apparatus described herein, and, most importantly, they modify slightly the calculated values of thermodynamic temperature given in reference [35]. The data on sample S-M were obtained both during warming and during cooling, while those on sample S-U were obtained only on cooling.

The length of sample S-M at 23.6 °C was found to be 2.528 388 cm, or 92,567.3 fringes of light at 546.2271 ran in vacuum. Some 45 fringe differences were obtained on this sample over the range −25 to +700 °C. Table 4 shows the experimental data used in obtaining the fringe differences *ΔN* that reflect the change in sample length between two stable temperatures, the fringe differences derived from the chosen fitting equation, and the differences between the experimentally derived values and the calculated values. The whole fringe differences occurring between the steady temperatures listed in column 2 were inferred from measurements made on other samples of the same alloy; the fractional fringe counts are given in column 3 of table 4. The fractional fringe values were corrected for the refractive index of the medium as obtained from the pressures given in column 4, thus obtaining the values given in colvunn 5.

**Figure 16:**
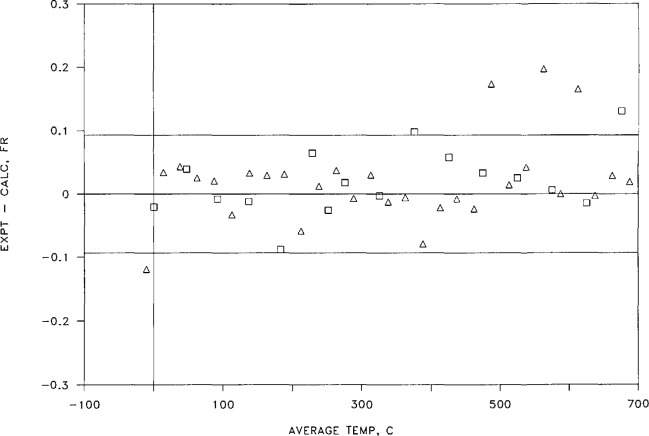
Differences in fringes of light at 546 nm between the length changes that were observed experimentally for sample S-M (80 Wt% Pt + 20 Wt% Rh) and those that were calculated from a 4th degree fitting equation (see table 5). Squares—data taken during cooling cycle. Triangles—data taken during warming cycle. All data points but one were obtained over at least a 20 °C temperature interval. Horizontal lines are drawn at the levels that indicate ± 1 ppm of the sample length.

The resulting values of *ΔN*_exp_. given in column 6, were fitted in the manner described above. The fitting parameters thus obtained are given in table 5. As one can see by examining table 5, there is little advantage in using a *t*^5^ term in the fitting procedure, and none in using a *t*^6^ term. The standard deviation of the four-term polynomial is somewhat smaller than 0.07 fringe, with the coefficients of the fitting equation as shown in table 5. Using these coefficients, one can obtain a set of *ΔN*_cai_ values for the experimental temperatures as given in column 7 of table 4. The deviations of the calculated data from the experimental data, listed in column 8, are shown in figure 16. No systematic differences appear between the data obtained during a cooling cycle and that obtained during warming.

It is clear from study of both table 5 and figure 16 that the newer apparatus provides linear thermal expansion values of excellent precision; 87% of the data points shown lie within ± 1 ppm of a four-term fitting equation.

The linear thermal expansion for sample S-M can be expressed in terms of eq (27), using the coefficients given in table 5 and the sample length derived for 0 °C,

$$\epsilon (t,0{}^{\circ}C)=8.707\times 10^{-6}t+2.177\times 10^{-9}t^{2}-1.037\times 10^{-12}t^{3}+5.729\times 10^{-16}t^{4}.$$ (29)

**Figure 17:**
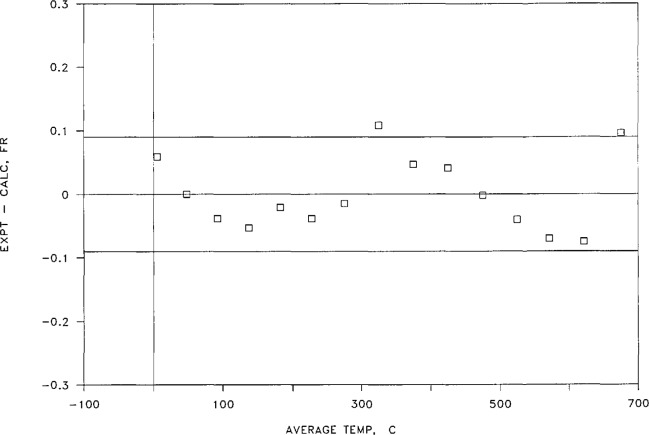
Differences in fringes of light at 546 nm between the length changes that were observed experimentally for sample S-U (80 Wt% Pt + 20 Wt% Rh) and those that were calculated from a 4th degree fittmg equation (see table 7). The data were obtained during one cooling cycle. Horizontal lines are drawn at the levels that indicate ± 1 ppm of the sample length.

A second sample of the same alloy was prepared from the NIST gas thermometer bulb in the same manner as sample S-M. Designated S-U, the sample’s bench-top (23.2 °C) length, expressed in fringes of light of 546.2271 nm, was measured as 90,445.6 fringes. The linear thermal expansion of sample S-U was measured in much the same way as S-M, although with fewer (15) steady temperatures. The results, however, were quite similar. Tables 6 and 7 show the information obtained from measurements on sample S-U, and figure 17 shows the differences between the experimental and calculated values. Again, most of the differences (13 of the 15 points) lie within ±1 ppm of the sample length.

The linear thermal expansion for sample S-U, using the coefficients from table 7 and the sample length calculated for 0 °C, is

$$\epsilon (t,0{}^{\circ}C)=8.730\times 10^{-6}t+2.056\times 10^{-9}t^{2}-7.999\times 10^{-13}t^{3}+4.111\times 10^{-16}t^{4}.$$ (30)

It is useful to examine the relationship between the linear thermal expansion coefficients determined a decade ago on a sample of 80 Wt% Pt + 20 Wt% Rh (see eq (10) in sec. 6.2 and reference [19]) and those of the two samples measured with the newer thermal expansion apparatus [eqs (29) and (30)]. Such a comparison can demonstrate the stability of the alloy to temperature cycling, and can show as well the practical similarity of two different thermal expansion facilities.

One method for comparing the three sets of results on the alloy in question would consist of a comparison of the thermal expansion coefficients obtained by fitting the separate sets of data. In table 8, we show the three sets of coefficients determined independently, along with the estimated standard deviations of the respective fits, normalized to unit sample length. Comparison of the three equations shows agreement within about two parts per million over the measured temperature range.

**Figure 18:**
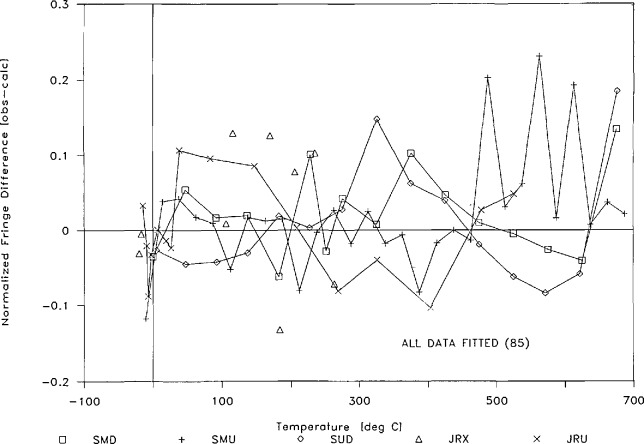
Differences in fringes of light at 546 nm, normalized to a uniform sample length, between the length changes observed experimentally and those that were calculated from eq (26) carried to the 4th degree, for three samples of 80 Wt% Pt + 20 Wt% Rh alloy considered as a single set of pooled data. Triangles and x’s—data from reference [19], obtained about a decade ago on a sample cut from a sheet of the stated composition. Triangles indicate data obtained in a more or less random sequence of heating and cooling; x’s indicate data taken in a single warming sequence. Squares and +’*s*—data obtained during cooling and warming, respectively, on sample S-M. Diamonds—data obtained during cooling on sample S-U. Both of the latter samples were cut from the NBS/NIST gas thermometer bulb following the completion of the gas thermometry experiments. One ppm of sample length corresponds to ±0.1 fringe.

The similarity of the three sets of measurements can be demonstrated more clearly by fitting eq (26) to all of the data as a single set. This we have done, after normalizing the three sets of fringe differences *ΔN(t_i_,t_i+_*_1_) to a uniform sample length. The thermal expansion coefficients thus determined for the pooled data are given in table 8, and the differences between the experimental and calculated values are shown in figure 18. One can see that no systematic difference larger than the imprecision of the fitting process is evident between any pair of samples. Furthermore, the estimated standard deviation of the pooled-data fit is quite comparable to those determined for the individual samples. These facts indicate that annealed samples of Pt-Rh alloy are extremely stable metallurgically, and that the two versions of the thermal expansion measurement apparatus are remarkably consistent in their performance.

The pooled-data thermal expansion equation,

$$\epsilon (t,0{}^{\circ}C)=8.70484\times 10^{-6}t+2.24455\times 10^{-9}t^{2}-1.2136\times 10^{-12}t^{3}+6.9642\times 10^{-16}t^{4},$$ (31)

will be used later to correct the observed gas thermometry results that were reported in reference [35].

In addition to the random uncertainty levels of ±2 ppm, there are systematic uncertainties in the thermal expansion determinations whose magnitudes can only be estimated. These include differences between the sample temperature and thermometer *T* (fig. 14), used to obtain the sample temperature, which were estimated as no larger than 0.001 °C uncertainty in the fringe counts, which were estimated as no larger than 0.01 fringe; uncertainty in the actual index of refraction of the gas that occupies the sample space, which was estimated to correspond to no more than 0.02 fringe; uncertainty in the vacuum wavelength of the spectrometer light source, which was estimated to be less than 1 ppm; and changes in the sample thermal expansivity or in expansion-related properties during the measurements, which were estimated as less than 2 ppm by virtue of the agreement shown in figure 18.

## 9. Scale Differences From 230 to 660 °C

As noted above, much of the gas thermometry data needed to calculate Kelvin thermodynamic temperature values in the range 230 to 660 °C was available in a laboratory microcomputer as soon as the measurements had been performed. Installation on the laboratory computer of the computational programs used earlier on the central NBS computer by Guildner and Edsinger [21] allowed the quick evaluation of the quantity *t*(KTTS)-*t*(IPTS-68) for a given measurement and thus the modification of procedures or apparatus as needed. The linear thermal expansion coefficients for sample S-M [eq (29)] were incorporated into the program to calculate the gas bulb volume at the various gas thermometer temperatures.

### 9.1 IPTS-68 Temperatures

The specially prepared PRT’s used in the high-temperature measurements were of sufficient quality to define the IPTS-68 by means of the usual calibration procedure. During the measurements, the thermometers were calibrated four times. One of the calibrations included measurements at the aluminum freezing-point temperature.

In use, the thermometers were re-calibrated at the triple point of water after each high-temperature run because of the possibility of drift as a result of mechanical shock or exposure to high temperatures.

IPTS-68 values of temperature near 660 °C were derived from the relation

$$t_{68}=t^{\prime }+0.045{}^{\circ}C,$$ (32)

where *t’* has the meaning given in the IPTS-68 text [48]. This definition, while not the standard one for this range of temperature, is more precise than the standard one based upon the Pt vs. (Pt + 10 Wt% Rh) thermocouple thermometer.

### 9.2 Non-Ideality of the Working Gas

Guildner and Edsinger used eq (20) to generate values of the second virial coefficient for ^4^He in the range 0 to 457 °C. A review of more recent literature on this topic [46] indicated to Edsinger and Schooley that eq (19) still was adequate to represent the second virial data, and that it also would suffice for generating values up to temperatures as high as 660 °C within uncertainty limits that range from ±0.2 cm^3^/mol at 0 °C to ±0.4 cm^3^/mol at 660 °C.

### 9.3 High-Temperature Drift

For the most part, the gas-thermometry measurement procedures and computations used in the most recent experiments were very similar to those described in section 7. The accuracy of the computational programs was verified by re-calculating results obtained earlier, using the original data. One change in procedure, however, is worth noting because it yielded puzzling results. This change involved an attempt to repeat the filling conditions after obtaining a pair of measurements at 0 °C and the test temperature.

In this study, the gas bulb was filled to ~13.3 kPa while it was maintained at 0 °C in the ice bath. After filling, the gas bulb was closed off and the gas thermometer was moved from the ice bath to the high-temperature furnace. The furnace was heated to a temperature in the range 230 to 660 °C, and the furnace controls were set so as to minimize the temperature gradients in the region of the gas bulb, making full use of the extra thermocouple thermometers arrayed along the capillary all the way down to the gas bulb. During the heating period, the height of the gage-block stack was changed, as usual, and the counterpressure was adjusted to track the calculated gas-bulb pressure within about 0.2 kPa. After measurements were completed at the upper temperature, the gas thermometer was returned to the ice bath and the manometer gage-block stack was returned to its original height.

**Figure 19:**
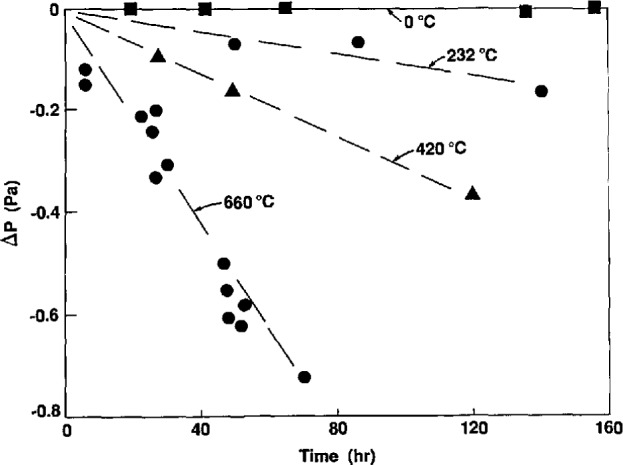
Temperature-dependent drift in tlie measured pressure of tlie NBS/NIST gas tliermometer, observed while maintaining the thermometer at various elevated temperatures. This effect was found while using a cylindrical Pt-Rh alloy gas-thermometer bulb with a thin (~ 1 mm) bottom plate. Subsequent measurements with a heavier (~2.5 mm) bottom plate showed approximately one-half as much drift at a particular temperature. The working pressure typically ranged from 13.3 kPa at 0 to 45 kPa at 660 °C. Note that no effect was seen at 0 °C.

This process, which typically lasted 7 d, invariably led to a final gas-bulb pressure that was lower than its starting value, the discrepancy being the larger, the higher the intervening furnace temperature and the duration of the high-temperature portion of the measurement. During one series of observations, the gas-bulb pressure was monitored while the gas thermometer was maintained for several days at various elevated temperatures. Typically, the observed drift amounted to −0.027 Pa/d at 231 °C, −0.067 Pa/d at 420 °C, and −0.27 Pa/d at 660 °C, as shown in figure 19. In terms of the thermodynamic temperature of the gas bulb, these quantities correspond to drifts of −0.54, −1.3, and −5.4 mK/d, respectively. No such drift in the gas-bulb pressure occurred while the gas thermometer was maintained at 0 °C, even for a period of several weeks.

There was no apparent explanation for this effect. The rather thin-walled (~ 1 mm) gas bulb was modified by replacing its bottom cap with thicker material (~2.5 mm), as noted in section 8.2, against the possibility that the pressure change had arisen from creep of the gas bulb, and subsequent gas-thermometry measurements of the type described above resulted in a high-temperature drift that, while still observable, was diminished by at least a factor of two at 660 °C.

The gas thermometry measurement procedure was modified in order to minimize, insofar as possible, the effect of the high-temperature drift mentioned above. The gas bulb was evacuated at a temperature at least as high as that which was to be measured; then the furnace was set at the upper measuring temperature. The gas bulb and manometer were filled with the desired quantity of freshly purified ^4^He, and both radial and vertical gradients in the furnace were carefully minimized (usually within ±0.003 °C). Then the gas thermometer was allowed to equilibrate. Following this step, the off-set in the capacitance diaphragm gage was measured and all power to the furnace heaters was turned off. An ice-bath measurement and thus a de-termination of *t*(KTTS)-*t*(IPTS-68) at the chosen high temperature could be accomplished in this fashion within 48 h of the time when the gas bulb was filled. This procedure avoided long periods of heating and, although it permitted the evaluation of only one IPTS-68 temperature in any one measurement, it minimized any systematic error in the results arising from the unexplained high-temperature drift. The maximum uncertainty from this source was estimated as -0.0006 °C for our measurements at 230 °C and as −0.006 °C for those at 660 °C.

It is interesting to note that Guildner and Edsinger [21] found no such drift during their studies. It was generally their practice to fill the gas bulb at the highest temperature to be studied, then to measure gas-bulb pressures at several temperatures in a decreasing sequence. They did not generally return to the filling temperature to demonstrate the accurate recovery of the filling pressure, but they found no drift during several occasions in which they left the gas bulb at the same temperature for several days.

### 9.4 Results and Their Uncertainties

The results of 26 determinations of *t*(KTTS)-*t*(IPTS-68), obtained in the manner just described, were presented by Edsinger and Schooley in reference [35] and are shown in figure 12. The value 660.342±0.015 °C was obtained for the thermodynamic temperature of the aluminum freezing point.

Four different filling pressures were used in measurements at 660 °C in an effort to verify the accuracy of the thermomolecular correction, which had been obtained from an experimental study conducted on a capillary different from the one used in the gas thermometry experiments (see sec. 7.3). This range of pressures required the employment of thermomolecular corrections as large as 0.3 °C at 660 °C. As can be seen in table 2 of reference [35], the value of the thermodynamic temperature determined in this sequence of measurements varied by no more than 0.0045 °C, indicating that the thermomolecular correction was known within a few percent.

A summary of the estimated uncertainties of the measurements comprising a gas-thermometer temperature determination are given in table 9. At 450 °C, the overall random uncertainty was given as 0.02 °C at the 99% confidence level [35]. By comparison, the corresponding uncertainty of the determination by Guildner and Edsinger [21] at the same temperature was given as no larger than 0.005 °C. Thus the difference between the two sets of determinations (approximately 0.03 °C) is slightly larger than the combined uncertainties.

After the gas thermometry measurements were complete to 660 °C, the gas bulb was disassembled from its protective case and several samples were cut from it for measurement of its thermal expansivity as described in section 8.5. X-ray analysis was used to verify that the entire gas bulb consisted of the same alloy composition.

## 10. Comparison of Gas Thermometer Results

The largest sources of uncertainty in the work of Guildner and Edsinger were ascribed to uncertainty in the knowledge of the non-ideality of the working gas and to uncertainty in the dead space correction, as shown in table 2. The uncertainty in the thermal expansion of the gas bulb was estimated as ±0.5 mK at the “one-sigma” level; in retrospect, we find that an alternative treatment of the thermal expansion data would result in a ±0.003 °C shift in the calculated thermodynamic temperature at 457 °C. Such a shift would bring the data shown in figure 12 into slightly closer agreement. We also ascribe a slightly larger uncertainty to the calculation of the gas imperfection correction (about ±0.0025 °C at 457 °C) based upon an assessment of the present literature. Finally, one can recall the composite nature of the gas bulb in the previous work, and the necessity to predict its effect upon the overall thermal expansion. While no details were given of this prediction, Guildner and Edsinger considered that the problem had negligible impact upon the accuracy of their results.

On the other hand, the experiments of Edsinger and Schooley were notable for the rather larger uncertainties in the determination of IPTS-68 temperatures, arising from the difficulty of setting and measuring the gas-bulb temperature in the gaseous environment of the high-temperature furnace. Also notable in the more recent experiments was the curious drift in the gas-bulb pressure (see fig. 19), an effect that cannot be ascribed to leakage in the equipment, or, indeed, to any unequivocal source. Taking account of the completed thermal expansion results given in section 8.5 modifies the published results of Edsinger and Schooley only slightly. Replacing the coefficients of eq (29), which were employed in calculating the gas-thermometry results given in reference [35], with the coefficients of eq (31) has the effect of increasing the calculated thermodynamic temperatures by 0.002 °C at 250 °C, by 0.0033 °C at 400 °C, and by 0.001 °C at 630 °C. While these corrections are not large ones, they illustrate the great sensitivity of gas thermometry measurements to the thermal expansion of the gas bulb itself.

In comparing the two sets of experimental results at 457 °C, as shown in figure 12, one finds very similar levels of experimental variation in the results, about 0.006 °C in reference [21] and 0.005 °C in reference [35]. The estimated imcertainties associated with the determinations of thermal expansion, of gas-bulb creep, of the gas-bulb temperature, and of the non-ideality of the working gas in the more recent work [35], however, are considerably larger than their earlier counterparts, resulting in overall random uncertainties estimated to range, at the level of one standard deviation, from 0.0045 °C at 230 °C to 0.008 °C at 660 °C. The combined uncertainties at the 99% confidence level nearly overlap both at 230 and at 457 °C.

## 11. Summary and Conclusions

The work described herein has been pursued over the course of several decades. It has achieved its goal of evaluating the accuracy of the international scale of temperature with respect to the basic Kelvin Thermodynamic Temperature Scale. The IPTS-68 was shown to deviate from the Kelvin Thermodynamic Scale by amounts far in excess of those expected when the international scale was promulgated, leading to a world-wide effort to perfect a replacement scale. In fact, this replacement scale, to be known as the International Temperature Scale of 1990 (ITS-90) is essentially complete at the time of this writing.

In the course of preparing the equipment needed for this project, a mercury manometer facility was constructed that was, and very likely remains to-day, the most accurate pressure-measurement facility in the world. Similar advances in the construction of capacitance level sensing equipment, in constant-volume valves and articulating seals, and in high-accuracy dilatometry have resulted from this project.

The basic physical concepts of the gas thermometry experiments described herein involve techniques that are well understood, while their realization and refinement have occupied highly trained and dedicated workers during whole careers of meticulous effort. The work described herein has considerably improved our understanding of the Kelvin Thermodynamic Temperature Scale, but it is clear from a glance at figure 12 that the Kelvin Thermodynamic Scale still is uncertain in the range upwards from 200 °C by about ±20 ppm. Reducing this uncertainty is a task that we leave to future thermometrists, who well may employ a different technology.

## Figures and Tables

**Table 1:** Estimated uncertainties associated with mercury manometer

Feature	Uncertainty
Angle of bottom boss with respect to the mercury cell.	3 *μ* rad
Co-planarity of level-sensing capacitor and its guard.	6.6 mPa
Maximum depression of meniscus.	0.013 mPa
Maximum effect of ripples on measured meniscus height.	0.13 mPa
Maximum effect of tilt of sensing capacitor on measured miniscus height.	0.13 mPa
Maximum difference in electrostatic attraction effect between upper and lower cells.	0.06 mPa
Maximum effect of tilt of base on measured mercury height.	0.13 mPa
Maximum uncertainty of mercury level resulting from capacitance bridge imbalance.	0.12 Pa
Maximum flexure of Invar base as a result of weight of gage blocks under upper cell.	13 nm/atm
Maximum angle of wrung gage-block joints.	1 *μ* rad
Maximum temperature drift in cellar.	2 mK/d
Maximum uncertainty in ratio of gage-block heights at two measured temperatures.	0.5 ppm
Maximum uncertainty in ratio of mercury densities at two measured temperatures.	0.5 ppm
Maximum uncertainty in ratio of hydrostatic heads in measuring arm of manometer at two measured temperatures.	0.3 ppm
Overall uncertainty as a pressure-ratio device, (99+% confidence level)	1.5 ppm
Note that, for the measurement of a single pressure, the uncertainties are larger:	
Maximum uncertainty in mercury density.	1 ppm
Maximum uncertainty in mercury height.	0.5 ppm
Maximum uncertainty in gravitational acceleration.	0.5 ppm
Maximum uncertainty in hydrostatic head depends upon the situation; if the gas-tube height is known to 1 cm and the temperature profile for helium gas is known to 0.5 K, then	0.3 ppm
Overall uncertainty as a pressure standard, (99+% confidence level)	2 ppm
“With helium gas, the manometer operates as an absolute measuring device over the range 10–130 kPa with an (uncertainty) of about 2 ppm, and as a ratio device with an (uncertainty) of about 1.5 ppm. (Further improvement in this range) depends upon improvement in the measurement of the density of mercury, the acceleration of gravity, and in length metrology” [5].	

**Table 2:** Estimated uncertainties of 1976 NBS gas thermometer

Feature	Uncertainty
Dead space	0.9 mK (C)
Thermal expansion	0.5 mK (B)
Pressure ratio	0.5 mK (A)
	0.4 mK (B)
Gas imperfection	1.2 mK (C)
Thermomolecular	Negligible

a Three classes of uncertainty were recorded, all in terms of “one standard deviation”:

A. Random errors.

B. Errors producing a constant bias but evaluable in terms of random errors of subsidiary experiments.

C. Systematic errors not evaluable in statistical terms.

**Table 3:** Scale differences at four reference temperatures

Point	*t*_68_, °C	(*t*_th_ − *t*_68_)> °C	Random, °C (3 s.d.)	Systematic, °C (3 s.d.)
Steam	100	−0.0252	±0.0018	±0.00054
Tin	231.9681	−0.0439	±0.0022	±0.0015
Zinc	419.58	−0.0658	±0.0028	±0.0028
	457	−0.0794	±0.0028	±0.0031

**Table 4:** Experimental data for sample S-M (80 Wt% Pt + 20 Wt% Rh) run 502, October–November 1987 *N*(23.6 °C)=92,567.3±0.5 fringes

1	2	3	4	5	6	7	8
Steady temp. no.	*t*(68), °C	F.F^a^.	Gas press. kPa	F.F. corr.	*ΔN*_exp_ b	*ΔN*_cal_	Dev fr^c^
1	700.086	0.559	0.169	0.557			
la					−50.154	−50.285	0.131
2	650.400	0.406	0.268	0.403			
2a					−50.071	−50.056	−0.015
3	600.202	0.334	0.184	0.332			
3a					−48.893	−48.899	0.006
4	550.449	0.442	0.275	0.439			
4a					−48.843	−48.868	0.025
5	500.009	0.601	0.392	0.596			
5a					−47.301	−47.334	0.033
6	450.451	0.297	0.149	0.295			
6a					−46.925	−46.983	0.058
7	400.551	0.372	0.127	0.370			
7a					−46.794	−46.892	0.098
8	350.007	0.582	0.411	0.576			
8a					−45.319	−45.316	−0.003
9	300.413	0.259	0.145	0.257			
9a					−44.504	−44.522	0.018
10	250.916	0.761	0.447	0.753			
10a					+0.948	+0.974	−0.026
11	252.007	0.705	0.211	0.701			
11a					−41.935	−42.000	0.065
12	204.567	0.769	0.136	0.766			
12a					−39.312	−39.224	−0.088
13	159.530	0.457	0.124	0.454			
13a					−38.989	−38.977	−0.012
14	114.001	0.469	0.155	0.465			
14a					−36.990	−36.982	−0.008
15	70.007	0.480	0.200	0.475			
15a					−37.054	−37.093	0.039
16	25.009	0.429	0.295	0.421			
16a					−40.308	−40.287	−0.021
17	−24.988	(0.115)^d^	0.060	(0.113)^d^			
18	−24.609	0.347	0.067	0.345			
18a					22.243	22.362	−0.119
19	+3.292	0.591	0.104	0.588			
19a					17.818	17.784	0.034
20	25.206	0.412	0.216	0.406			
20a					20.890	20.847	0.043
21	50.607	0.304	0.287	0.296			
21a					20.272	20.247	0.025
22	74.999	0.576	0.323	0.568			
22a					21.002	20.981	0.021
23	100.005	0.575	0.231	0.570			
23a					21.058	21.091	−0.033
24	124.882	0.628	0.016	0.628			
24a					21.991	21.958	0.033
25	150.521	0.620	0.039	0.619			
25a					21.024	20.995	0.029
26	174.802	0.646	0.165	0.643			
26a					22.026	21.995	0.031
27	200.007	0.673	0.227	0.669			
27a					21.803	21.862	−0.059
28	224.836	0.474	0.119	0.472			
28a					23.032	23.020	0.012
29	250.752	0.506	0.129	0.504			
29a					21.969	21.932	0.037
30	275.236	0.477	0.216	0.473			
30a					23.020	23.027	−0.007
31	300.734	0.495	0.113	0.493			
31a					22.124	22.094	0.030
32	325.007	0.620	0.209	0.617			
32a					22.916	22.929	−0.013
33	350.005	0.537	0.243	0.533			
33a					23.105	23.111	−0.006
34	375.011	0.643	0.333	0.638			
34a					23.811	23.890	−0.079
35	400.666	0.450	0.045	0.449			
35a					23.210	23.232	−0.022
36	425.430	0.660	0.080	0.659			
36a					23.011	23.019	−0.008
37	449.793	0.672	0.133	0.670			
37a					23.950	23.974	−0.024
38	474.986	0.622	0.173	0.620			
38a					24.812	24.638	0.174
39	500.689	0.433	0.119	0.432			
39a					24.067	24.053	0.014
40	525.601	0.501	0.184	0.499			
40a					23.980	23.938	0.042
41	550.220	0.481	0.220	0.479			
41a					24.861	24.663	0.198
42	575.404	0.344	0.355	0.340			
42a					24.260	24.260	0.000
43	600.000	0.602	0.148	0.600			
43a					25.008	24.842	0.166
44	625.006	0.611	0.267	0.608			
44a					25.002	25.005	−0.003
45	649.993	0.611	0.128	0.610			
45a					24.987	24.958	0.029
46	674.750	0.599	0.168	0.597			
46a					25.832	25.813	0.019
47	700.162	0.431	0.185	0.429			

a F.F.; fractional fringe count at the fiducial mark.

b *ΔN*_exp_ corrected differences in sample length, expressed in fringes.

c Dev; *ΔN*_exp_ − *ΔN*_cal_. These differences are plotted in figure 16.

d The experimental uncertainty of this value was unusually high.

**Table 5:** Fitting parameters for the thermal expansion of sample S-M (80 Wt% Pt + 20 Wt% Rh)

Equation used in least-squares fitting procedure:			
$$\Delta N_{\text{vac}}(t_{i},t_{i+1})=\sum_{n=1}^{m}A_{n}({t^{n}}_{i+1}-{t^{n}}_{i})$$ (26)			
Determination of number of polynomial terms for “best fit”:			
*p* = 45 data points			

*m*	*σ* a	*F*_m_ b	*F*_0.95_(1, *p-m*)

1	2.169 2		
2	0.130 7	12073.4	4
3	0.084 7	60.4	4
4	0.066 7	26.6	4

5	0.061 8	7.8	4
6	0.060 4	2.9	4

Coefficients of 4th degree polynomial:		
*n*	*A_n_*	*σ* of *A_n_*

1	0.805 840 5	0.000 897 4
2	0.000 201 509	0.000 005 967
3	−0.000 000 095 958	0.000 000 014 034
4	0.000 000 000 053 023	0.000 000 000 010 233
*N*(0 °C)=92,548.2 fringes (calc)		

a *σ*: the estimated standard deviation of the fit.

b *F_m_*: an index used to estimate the level of significance of the *m*th coefficient in the equation fitted to the data.

$F_{m}=1+(p-m+1)[{\left(\sigma_{m-1}/\sigma_{m}\right)}^{2}-1]$ where *p* is the number of data points and *m* is the number of coefficients in the polynomial. The coefficient *A_m_* is considered significant at the desired level of confidence *F_m_* is greater than the corresponding value given in the fourth column. (Entries in column 4 were taken from a standard table for the *F*-distribution, e.g., table 3 of reference [34].)

**Table 6:** Experimental data for sample S-U (80 WT% Pt + 20 Wt% Rh) run 501, April-July 1987 *N*(23.2 °C)=90,445.6±0.5 fringes

1	2	3	4	5	6	7	8
Steady temp. no.	*t*(68), °C	F.F^a^.	Gas press. kPa	F.F. corr.	*ΔN*_exp_ b	*ΔN*_cal_	Dev fr^c^
1	700.801	0.482	0.305	0.479			
la					−49.867	−49.963	0.096
2	650.201	0.615	0.331	0.612			
2a					−55.118	−55.043	−0.075
3	593.602	0.498	0.348	0.494			
3a					−41.874	−41.804	−0.070
4	550.005	0.624	0.363	0.620			
4a					−47.081	−47.041	−0.040
5	500.298	0.543	0.372	0.539			
6	500.305	0.500	0.424	0.495			
6a					−46.961	−46.959	−0.002
7	449.976	0.539	0.424	0.534			
7a					−45.916	−45.957	0.041
8	400.004	0.624	0.437	0.618			
8a					−44.994	−45.041	0.047
9	350.301	0.630	0.435	0.624			
9a					−44.257	−44.366	0.109
10	300.594	0.374	0.427	0.367			
11	300.608	0.309	0.613	0.299			
11a					−44.073	−44.058	−0.015
12	250.454	0.236	0.597	0.226			
12a					−39.848	−39.810	−0.038
13	204.404	0.389	0.596	0.378			
14	204.405	0.483	1.243	0.460			
14a					−37.801	−37.780	−0.021
15	160.001	0.684	1.200	0.659			
15a					−38.122	−38.069	−0.053
16	114.503	0.563	1.147	0.537			
16a					−36.207	−36.169	−0.038
17	70.505	0.358	1.093	0.330			
17a					−36.744	−36.744	0.000
18	24.954	0.616	1.028	0.586			
19	24.951	0.649	1.040	0.618			
19a					−31.368	−31.427	0.059
20	−14.766	0.264	0.408	0.250			

a F.F.; fractional fringe count at the fiducial mark.

b *ΔN*_exp_; corrected differences in sample length, expressed in fringes, that accompany changes from one steady-state temperature to the next.

c Dev; *ΔN*_exp_ − *ΔN*_cal_. These differences are plotted in figure 17.

**Table 7:** Fitting parameters for the thermal expansion of sample S-U (80 Wt% Pt+20 Wt% Rh)

Equation used in least-squares fitting procedure:			
$$\Delta N(t_{i},t_{i+1})=\sum_{n=1}^{m}A_{n}({t^{n}}_{i+1}-{t^{n}}_{i})$$ (26)			
Determination of number of polynomial terms m for “best fit”:			
*p = 15* data points			

*m*	*σ* a	*F*_m_ b	*F*_0.95_(1, *p-m*)

1	2.898 0		
2	0.138 9	6081.2	4.67
3	0.075 8	31.7	4.75
4	0.058 1	9.4	4.84

5	0.026 4	43.4	4.96
6	0.020 5	7.6	5.12

Coefficients of 4th degree polynomial:		
*n*	*A_n_*	*σ* of *A_n_*

1	0.789 424 4	0.001 161 2
2	0.000 185 896	0.000 007 379
3	−0.000 000 072 335	0.000 000 016 695
4	0.000 000 000 037 180	0.000 000 000 011 889
*N*(0 °C)=90,427.2 fringes (calc)		

a *σ*: the estimated standard deviation of the fit.

b *F_m_*: an index used to estimate the level of significance of the mth coefficient in the equation fitted to the data.

$F_{m}=1+(p-m+1)[{\left(\sigma_{m-1}/\sigma_{m}\right)}^{2}-1]$ where *p* is the number of data points and *m* is the number of coefficients in the polynomial. The coefficient *A_m_* is considered significant at the desired level of confidence *F_m_* is greater than the corresponding value given in the fourth column. (Entries in colimin 4 were taken from a standard table for the F-distribution, e.g., table 3 of reference [34].)

**Table 8:** Linear thermal expansion coefficients determined for three samples of 80 Wt% Pt+20 Wt% Rh

Sample^a^	*t*^1^ coef.	*t*^2^ coef.	*t*^3^ coef.	*t*^4^ coef.	*σ*_fit_ b
Ref [19] (25)	8.674 × 10^−6^	2.538 × 10^−9^	−2.081 × 10^−12^	1.480 × 10^−15^	0.062
S-M (45)	8.707 × 10^−6^	2.177 × 10^−9^	−1.037 × 10^−12^	5.729 × 10^−16^	0.073
S-U (15)	8.730 × 10^−6^	2.056 × 10^−9^	−7.999 × 10^−13^	4.111 × 10^−16^	0.065
Pooled (85)	8.705 × 10^−6^	2.245 × 10^−9^	−1.214 × 10^−12^	6.964 × 10^−16^	0.074

a Number of data points in parentheses.

b Estimated standard deviation of the 4th-degree fit normalized to a uniform sample length.

**Table 9:** Estimated uncertainties of gas thermoraetry results from 230 to 660 °C

Source	Type^a^	Magnitude	
		230 °C	660 °C
Manometer pressure ratio accuracy	C	±0.001	±0.0015
Gas bulb thermal expansion/creep	S	−0.0006	−0.006
Gas bulb thermal expansion measurements	R	±0.003	±0.006
Gas-bulb thermostat temperature inhomogeneity	R	±0.003	±0.005
PRT resistance-temperature relation	R	±0.001	±0.002
^4^He piuity	S	Unknown	
^4^He virial correction	**S**	±0.001	±0.004
Thermomolecular pressure correction	**S**	<0.001	<0.001
Capacitance diaphragm gage imbalance	C	±0.0002	±0.0002

a S=Systematic; R=Random; C=Combination.

## Footnotes

1 Certain commercial equipment, instruments, or materials are identified in this paper to specify adequately the experimental procedure. Such identification does not imply recommendation or endorsement by the National Institute of Standards and Technology, nor does it imply that the materials or equipment identified are necessarily the best available for the purpose.
